# Escape behaviors in prey and the evolution of pennaceous plumage in dinosaurs

**DOI:** 10.1038/s41598-023-50225-x

**Published:** 2024-01-25

**Authors:** Jinseok Park, Minyoung Son, Jeongyeol Park, Sang Yun Bang, Jungmoon Ha, Hyungpil Moon, Yuong-Nam Lee, Sang-im Lee, Piotr G. Jablonski

**Affiliations:** 1 School of Biological Sciences, Seoul National University, Seoul, South Korea; 2 School of Earth and Environmental Sciences, Seoul National University, Seoul, South Korea; 3 Department of Earth and Environmental Sciences, University of Minnesota, Minneapolis, MN, USA; 4 Department of Mechanical Engineering, Sungkyunkwan University, Suwon, South Korea; 5 Department of New Biology, DGIST, Taegu, South Korea; 6 Museum and Institute of Zoology, Polish Academy of Sciences, Warsaw, Poland

**Keywords:** Palaeontology, Evolution, Palaeoecology

## Abstract

Numerous non-avian dinosaurs possessed pennaceous feathers on their forelimbs (proto-wings) and tail. Their functions remain unclear. We propose that these pennaceous feathers were used in displays to flush hiding prey through stimulation of sensory-neural escape pathways in prey, allowing the dinosaurs to pursue the flushed prey. We evaluated the escape behavior of grasshoppers to hypothetical visual flush-displays by a robotic dinosaur, and we recorded neurophysiological responses of grasshoppers’ escape pathway to computer animations of the hypothetical flush-displays by dinosaurs. We show that the prey of dinosaurs would have fled more often when proto-wings were present, especially distally and with contrasting patterns, and when caudal plumage, especially of a large area, was used during the hypothetical flush-displays. The reinforcing loop between flush and pursue functions could have contributed to the evolution of larger and stiffer feathers for faster running, maneuverability, and stronger flush-displays, promoting foraging based on the flush-pursue strategy. The flush-pursue hypothesis can explain the presence and distribution of the pennaceous feathers, plumage color contrasts, as well as a number of other features observed in early pennaraptorans. This scenario highlights that sensory-neural processes underlying prey’s antipredatory reactions may contribute to the origin of major evolutionary innovations in predators.

## Introduction

The early function of pennaceous feathers remains unclear. Over the past 3 decades, spectacular dinosaur fossils with diverse feather types have been discovered^1^. Among these fossils, pennaceous feathers, the type of feathers that is adaptively modified for flying in modern birds, are limited to Pennaraptora^2^. The earliest pennaceous feathers were present on the distal forelimbs as small ‘proto-wings’ and around the tip of the tail as distal caudal plumage in the early-diverging pennaraptorans, as preserved in *Caudipteryx*^3^. Proto-wings were too small to be used for powered flight^4^. The functions of proto-wings and caudal plumage might have been related to foraging/hunting [insect netting^5,6^; pouncing on prey^7^; leaping for prey^8^; immobilizing large prey^9^; running while flapping^10,11^], or other behaviors [brooding^12^; wing-assisted incline running^13^; gliding^14^; intraspecific displays^15,16^]. Here, we evaluate a recently proposed hypothetical function of proto-wings and caudal plumage in dinosaurs^17^: the use of feathers to flush the prey and to pursue the flushed prey in a manner similar to the flush-pursue foraging strategy known in extant birds^18–20^.

**Figure 1:**
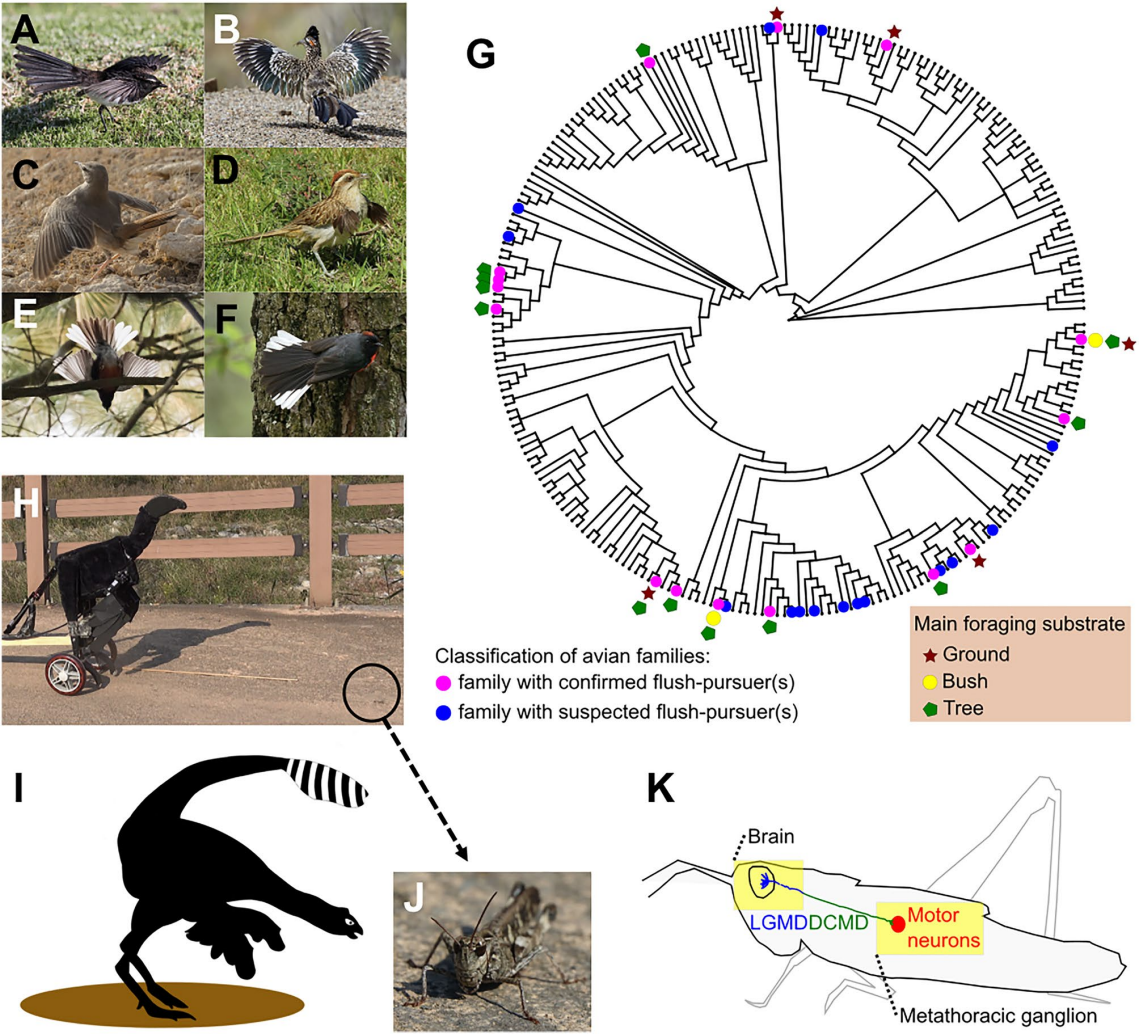
Diversity of avian flush-pursuers, prey with simple neural escape pathways, and the *Caudipteryx* robot used in behavioral experiments. (**A**–**F**) Examples of flush-pursuers (more examples in Text S1 linked to Fig. S1): *Rhipidura leucophrys*, *Geococcyx californianus*, *Cercotrichas galactotes*, *Tapera naevia*, *Myioborus pictus*, *Myioborus miniatus*, respectively. We used the following recordings from the Macaulay Library at the Cornell Lab of Ornithology: ML205494131, ML98307051, ML366333971, ML278048021, ML272399001, ML253368241. (**G**) Distribution of flush-pursuers among 248 avian families, showing that this strategy is widespread across diverse avian taxa (see Fig. S1 and Text S1 for more details); a quantitative phylogenetic analysis is a subject of a separate paper. (**H**) The robotic dinosaur (“Robopteryx”) in the natural habitat placed in front of a grasshopper. (**I**) Artistic depiction of the hypothetical flush-display by a feathered dinosaur. (**J**) *Oedaleus infernalis*, the species used in the experiments. (**K**) Looming-detecting neurons (LGMD/DCMD) involved in triggering escape responses to visual stimuli in orthopterans. © by Authors.

The avian flush-pursue foraging strategy involves visual displays of contrasting plumage on spread/flicked wings and tails (Fig. 1A–F, Fig. S1; Text S1). These displays trigger prey to escape from their hiding places, becoming available for aerial or cursorial pursuits and subsequent capture by predators^18,21^. This foraging strategy is based on the “rare enemy effect” concept within multispecies predator–prey systems^22^, where rare predatory strategies exploit antipredatory adaptations of prey against common predatory strategies. Flush-pursuers comprise a small proportion of avian guilds^18^ and are able to exploit visually triggered escape responses in prey. These responses are adaptations to avoid capture by typical non-flush-pursuers. The flush-pursue strategy is present in diverse avian clades, and their phylogenetic distribution (Fig. 1A–G, Fig. S1) indicates that this strategy evolved multiple times convergently. While the details of the flush-pursue adaptations differ among the different avian clades and must be assessed separately in each group, this strategy exploits the properties of prey’s relatively simple neural circuits that mediate escape responses to visual stimuli^18–20,23^, and is especially predicted when prey cannot precisely evaluate the absolute distance, size, speed, and type of the approaching predator^20^. Visual displays by flush-pursuing birds are found in various families of primarily insectivorous or omnivorous birds (Fig. 1A–G, Fig. S1; Text S1), and the adaptations to flush-pursue foraging may account for adaptive morphological divergence among subspecies of *Myioborus* flush-pursuers^24^. As the flush-pursue foraging may play a role in the evolutionary diversification of birds, it is possible that this foraging style also played a role in the diversification of pennaraptoran dinosaurs.

Escape behaviors in response to visual stimuli occur in many prey animals^23^: orthopterans^25^, flies^26^, crabs^27,28^, crayfish^29^, and small mammals^30^—taxa that pennaraptoran dinosaurs likely also hunted^31^. In addition to avian visual flush-pursuers, predator flush-pursue strategies based on visual flush displays and auditory or tactile flush-cues have been observed in several predator–prey systems, such as star-nosed moles and earthworms^32^, cheetahs and antelopes^33^, or squids and shrimps^34^. Finally, some bird species use non-visual cues to flush prey in order to pursue it (Text S1). Therefore, it is reasonable to consider that the flush-pursue strategy might have also occurred in the multispecies ecological systems of predatory dinosaurs and their prey.

The flush-pursue hypothesis (Hypothesis 1 in Table 1) incorporates several earlier hypotheses (Hypotheses 2–6 in Table 1) and involves three consecutive elements, with two elements being shared with previously proposed hypotheses: (element 1) using visual flush-displays with feathered forelimbs/tails to trigger escapes in prey; (element 2) using feathered forelimbs/tails for generating drag and/or lift during prey pursuits (e.g., the “running while flapping” hypothesis^10,11^, the “leaping” hypothesis^8^) or for attacking flushed prey immediately after landing (similar to the “pouncing on prey” hypothesis^7^); (element 3) using quick forward head movements, facilitated by a long neck, or possibly the use of hindlimbs (or even forelimbs) for prey capture, which could be aided by proto-wings functioning as insect nets^6^ or immobilizing prey^9^. The hypothesis highlights a positive feedback loop between the “flush (element 1)” and the “pursue-attack” (elements 2 and 3) elements of the strategy. The use of plumage to flush prey could have increased the frequency of chases after escaping prey, thus amplifying the importance of plumage in drag-based or lift-based maneuvering for a successful pursuit. This, in turn, could have led to the larger and stiffer feathers for faster movements and more pronounced visual flush-displays. These adaptations may have further intensified the visual flush-displays, ultimately promoting foraging based on flushing, pursuing, and capturing the flushed prey.

The contrast in plumage enhances the flush-pursuit foraging efficiency of extant flush-pursuers^18–20,35^ and could have had a similar effect in pennaraptoran dinosaurs. In addition, plumage color patterns in non-avian feathered dinosaurs might have also played a role in many aspects of their life, including signaling functions, thermoregulation, and crypsis^15,36,37^, just as it happens in extant avian flush-pursuers who often use wing/tail displays in aggressive or antipredatory contexts. Some extant flush-pursuers, such as the Painted Redstart (*Myioborus pictus*), use white patches both in flushing prey^35,38^ and in territorial interactions. It is conceivable that plumage coloration in pennaraptorans, such as the light and dark regions in the tail fan of *Caudipteryx*^3^, might also have been used in inter- and intra-specific displays and communication^15,16,39,40^, regardless of whether it was used for flush-pursuit or not. Among the hypothetical functions, the flush-display function can be assessed by testing extant arthropod prey with escape circuits representing a possible ancient prey for pennaraptorans.

**Figure 2:**
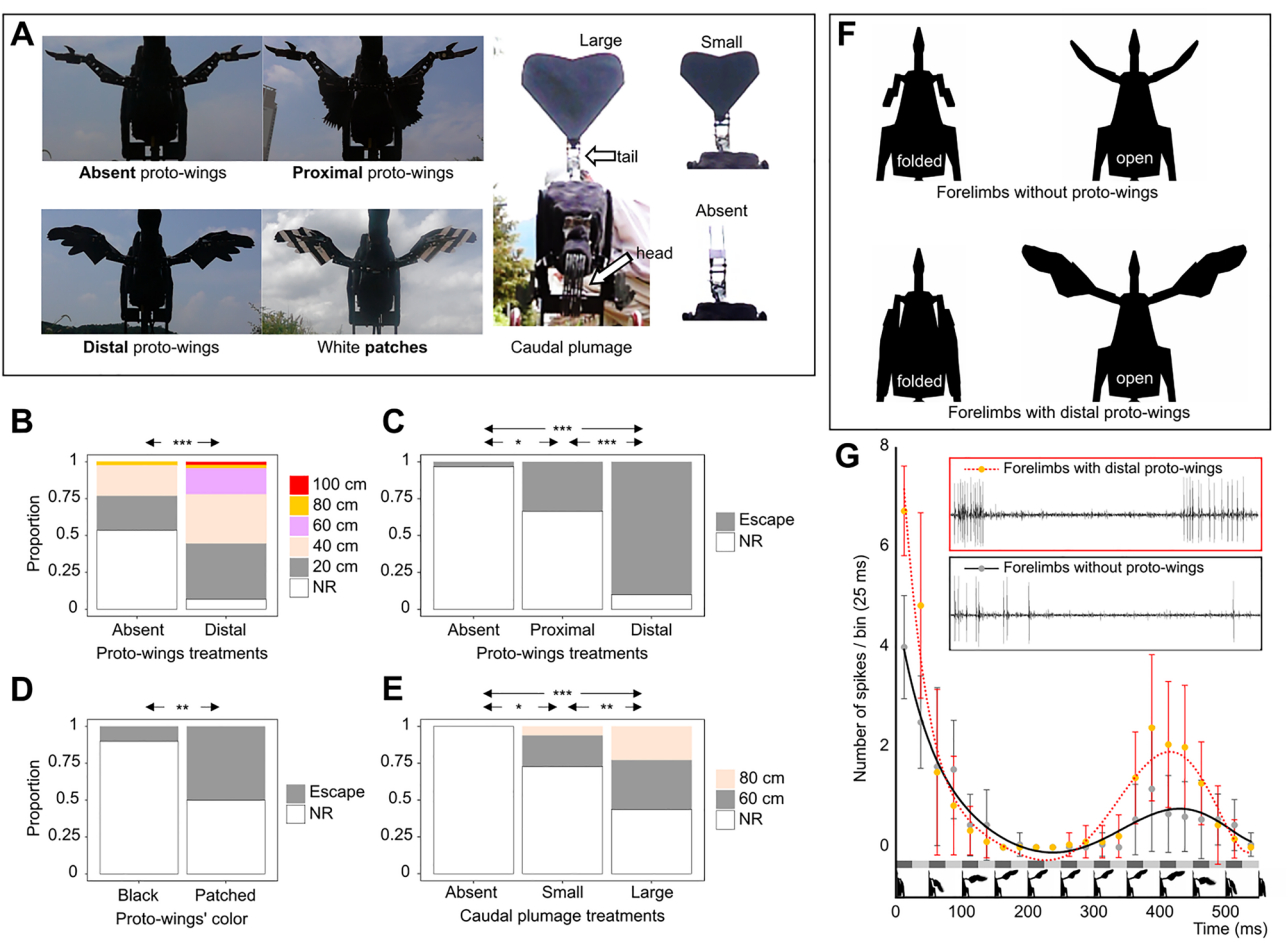
Behavioral and neurophysiological experiments. (**A**) Experimental treatments in behavioral tests. (**B**) Effect of the presence of the proto-wings on the escape distance and frequency of grasshoppers during a stepwise approach of the robot. The robot started from a distance of 100 cm, with up to five stops with displays at 100, 80, 60, 40, and 20 cm from the grasshopper and ended at 20 cm or when the grasshopper escaped (Methods Part 4 Experiment 1; details in Table S1). (**C**–**E**) Effects of the presence and location of proto-wings (C), their color (D), and the presence and size of caudal plumage (E) on the escape frequency of grasshoppers (Methods Part 4 Experiments 2, 3, 4; Table S2–S4). Statistical differences were tested using Dunn’s test with Bonferroni correction (**B**,**E**), Chi-square test with Bonferroni correction (**C**), and Chi-square test with Yates’ continuity correction (**D**). NR means “no response.” * indicates *P* < 0.05; ** indicates *P* < 0.01; *** indicates *P* < 0.001. (**F**) Schematic representation of the experimental treatments in neurophysiological experiments (the first frame and the frame with the most extended forelimbs of the animation). (**G**) Firing rate of the grasshopper’s LGMD/DCMD escape pathway (average ± SD; n = 18, i.e., six recordings from each of 3 individuals; recordings for each individual are shown in Fig. S3B–G) in response to animations with (dotted orange line; Video S1, part 4) and without (solid black line; Video S1, part 5) distal proto-wings. Gray bars represent bins, and screenshots from the animation with proto-wings are shown at 50 ms intervals along the horizontal axis. The two insets represent examples of recorded responses (additional details in Fig. S3; Tables S5–S6; Methods Part 5).

**Figure 3:**
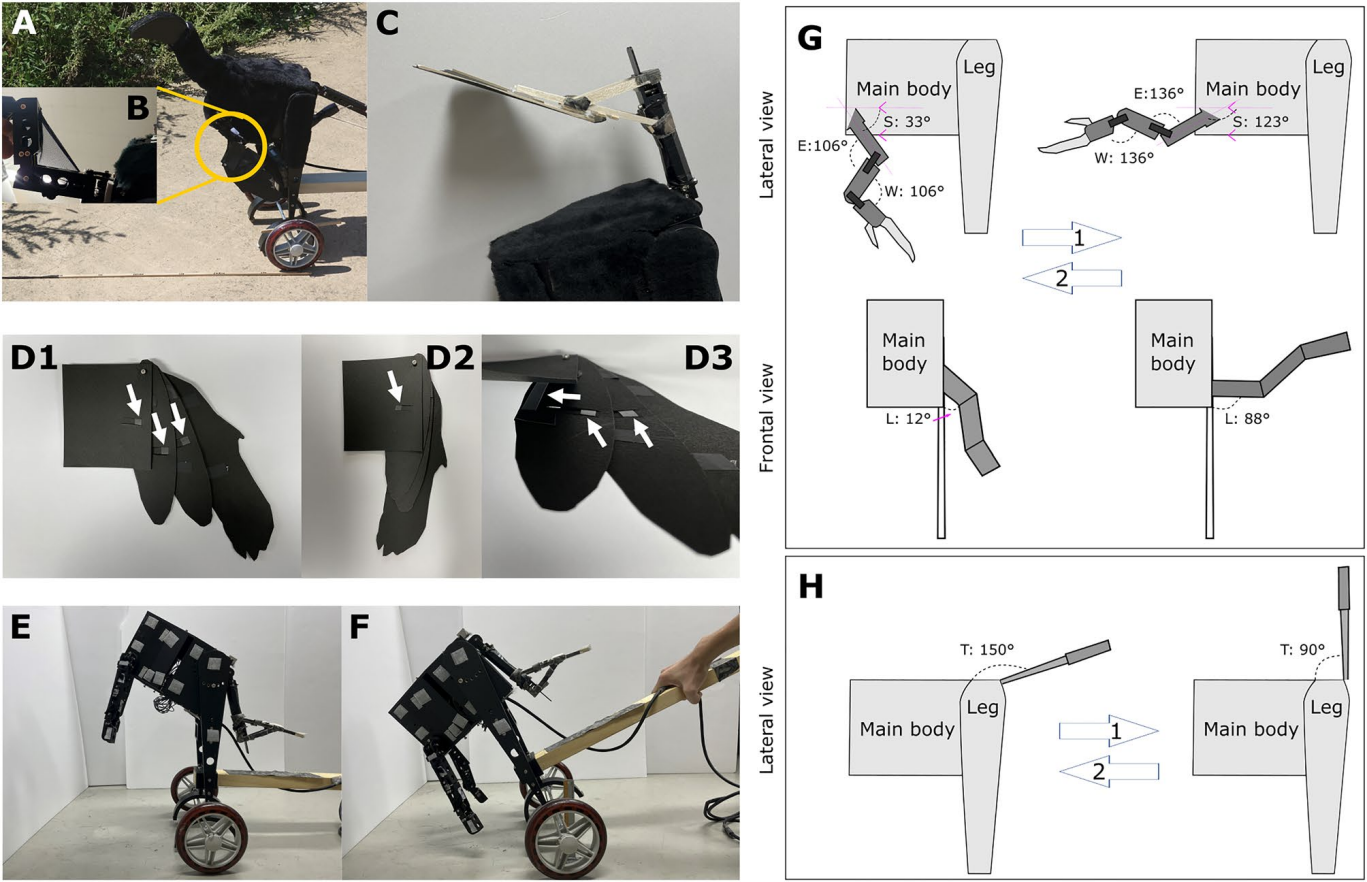
Details of the *Caudipteryx* robot (“Robopteryx”) and its movements. (**A**) The metal casing of the robot was covered with black felt. The head was made from black polystyrene. (**B**) Dark stockings were used to imitate the propatagium. (**C**) An additional structure was attached to the tail to imitate a bending tail. (**D1**–**D3**) The distal proto-wing was made using black paper. Arrows indicate plastic stoppers adjusting the minimum (D2) and maximum (D1) range of proto-wing expansion movements. (**E**) For experiments concerning proto-wings (Experiments 1 to 3), the robot’s main body was tilted 37° upward to imitate a flush-pursuer position, similar to observed ground-foraging flush-pursuers like the Greater Roadrunner (link 1 in Text S1). (**F**) In the experiment concerning caudal plumage (experiment 4), the robot’s main body was tilted 40° downward to imitate a situation in which the upward movements of the tail might potentially affect the grasshopper; otherwise, that tail is behind the body and not visible to the grasshopper. This posture is observed in birds when they focus on a specific location in front of them. (**G**) Lateral (upper row) and frontal (lower row) view on the angles defining the range of movements of the robot’s forelimbs. The flushing movement starts from the resting posture of the forelimb (33° in S, 106° in E, 106° in W, 12° in L) and within 0.42 s reaches (arrow 1) the maximum posture (123° in S, 136° in E, 136° in W, 88° in L), pauses for 0.2 s, and then reverts back within 0.42 s (arrow 2) to the resting posture. (**H**) Lateral view of the tail movements; during 0.33 s the robot lifts its tail (arrow 1) from T = 150° to T = 90°, and then lowers it (arrow 2) back to T = 150° during 0.33 s.

The flush-displays that cause an increase in the frequency and/or distance at which prey initiates escapes invariably lead to improved foraging efficiency by avian flush-pursuers^18–20^. Therefore, the flush-pursue hypothesis can be experimentally evaluated by testing the “flush” function of the distal proto-wings, distal caudal plumage, and their contrasting coloration on the escape reactions of prey organisms. Here, we used a robot (named Robopteryx; Figs. 1H, 2A, 3, Fig. S2) based on the morphology and size of the well-documented pennaraptoran dinosaur, *Caudipteryx*, to flush grasshoppers (Orthoptera, representing an ancient order of possible prey of pennaraptoran dinosaurs; Fig. 1J) by hypothetical dinosaurian visual displays (Fig. 2A). The robot represents a general cursorial bipedal theropod with a long tail and forelimb movement range similar to that of early pennaraptoran dinosaurs (Fig. 1H,I). We also recorded the responses of the LGMD/DCMD neural pathway (Fig. 1K), which is involved in orthopterans’ visually evoked jump-escape reactions, to computer animations of the hypothetical flush-displays (Fig. 2F) by pennaraptoran dinosaurs. Moreover, considering that the higher peak value of DCMD firing rate is an indicator of the higher probability that an escape-jump is triggered in a grasshopper by a looming visual stimulus^41^, we determine the effect of the presence of proto-wings on the peak DCMD firing rate. All these experiments are not aimed at providing evidence for homologous functions and behaviors between living birds and extinct pennaraptorans. Instead, our experiments are based on an analogy between avian species and putative pennaraptoran flush-pursuers. We believe that this approach can be instructive for proposing and further investigating hypotheses.

**Table 1:** Comparison of the flush-pursue hypothesis with previously proposed hypotheses.

Hypothesis descriptions: Brief descriptions of hypotheses aiming to explain the function of proto-wings and caudal plumage in feathered dinosaurs, especially during the initial stages of evolution in basal pennaraptoran theropods	Predictions: predictions of hypotheses regarding the plumage location and color, as well as the hypothetical adaptive functions of pennaceous feathers in feathered dinosaurs from basal pennaraptoran theropods with relatively small proto-wings and caudal plumage to more derived cursorial taxa with more developed proto-wings and caudal plumage				Consistency between basal Pennaraptora and predictions We evaluate whether the observed characteristics of basal pennaraptoran theropods align with the predictions made by the hypothesis [Y/N/x; stand for Yes/No/Irrelevant]. We narrowed down the scope to only the most basal taxa of pennaraptoran theropods, allowing us to focus specifically on the initial evolution of proto-wings and caudal plumage						
	P1: Location of the proto-wings and sex dimorphism (SD)	P2: Presence of the caudal plumage and sex dimorphism (SD)	P3: Bright patches on the forelimbs and tail feathers and sex dimorphism (SD)	P4: Predicted functions/traits/evolutionary trends of forelimbs (F:), neck/head (N:), hindlimbs (H:), and tail (T:) that benefit the hypothetical mechanism							
					P1	P2	P3	P4(F)	P4(N)	P4(H)	P4(T)
Mechanisms relevant to foraging: Hypothesis 1. Flush-pursue (FP) hypothesis^17^: It involves three consecutive elements; (i) visual flush-displays using feathered forelimbs/tails; (ii) the use of feathered forelimbs/tails for generating drag and/or lift during the pursuit of prey or for attacking flushed prey after it lands on a substrate; (iii) the use of rapid head movements on a long neck as extant birds do. Alternatively, the use of hindlimbs or even forelimbs for capturing prey, possibly aided by the use of proto-wings as insect nets or to immobilize the prey in the final stages of pursuit	Distal; no SD	Yes; no SD	Yes; no SD	F: Increase the surface area for more substantial display and for better assistance in motor control during the pursuit and capture of prey, especially during their escape flights, jumps, or immediately after landing. Within the anatomical constraints of early pennaraptorans, the forelimb movements serve as a sufficient looming display. But further development involving more pronounced folding and greater expansion of the forelimbs offers benefits for enhancing the “flush” element of the FP strategy. This would increase the size contrast between the displayed and non-displayed areas. Moreover, the development of stronger forelimb musculature, enabling lifting/spreading and folding/closing of the forelimbs, is advantageous, especially for drag-based motor control during the “pursue” element of the FP strategy N: Long-neck with skeleton/musculature that permits rapid forward movement during a prey strike enhances the efficiency of the final stages of pursuit. A visual system and inner ear promote visuomotor coordination, while the morphology of jaws or beaks facilitates precise striking and grasping of prey H: Hindlimb that aids in fast-running locomotion during pursuits of flushed prey can improve the efficiency of FP foraging T: Long-tail with a distal surface offers advantages for FP foraging. A spread feathered tail, capable of moving forward above the body or sideways, serves as a vigorous display and better assists in motor control during pursuits and captures of prey, especially during their escape flights, jumps, or immediately after landing	Y	Y	Y	Y	Y	Y	Y
Hypothesis 2. Flapping proto-wing hypothesis (running while flapping^10,11^): This hypothesis suggests that distal proto-wings were used to generate weak lift, possibly aiding in running, as demonstrated in a study involving robotic *Caudipteryx*. This behavior might have also been employed during the pursuit of prey. However, as the actual range of forelimb movement in real *Caudipteryx* may have been narrower than what was tested with the robot, hypothetical benefits during running might have been small in basal pennaraptoran theropods	Distal; no SD	Irrelevant; no SD	Irrelevant	F: Increase the surface area for better assistance in motor control during flapping; the powerful muscle, connected to the humerus, for sufficient stability in flapping. Efficient flapping requires the distal proto-wings to generate a strong power stroke^7^. Increasing the range of forelimb movements would benefit the “flapping proto-wing” mechanism. Those benefits may be relatively minor for small proto-wings with a restricted range of movements N: Irrelevant H: Hindlimb that aids in cursorial locomotion T: long and stiff tail assists in motor control (balance and quick turns) during running	Y^a^	x	x	Y	x	Y	Y
Hypothesis 3. Leaping (for prey)^8^: The core of this hypothesis is the use of proto-wings to generate lift, facilitating jumps or leaps towards flying prey and assisting in landing. Additionally, it involves using the tail for body balance control during a jump. If the pennaceous plumage surfaces are relatively small, the direct effect on leap trajectory is expected to be small. But the effect on body orientation (pitch and roll) during leaps still remains noticeable and may increase the foraging efficiency as evaluated in theoretical calculations^8^	Distal; no SD	Yes; no SD	No; higher crypticity is expected for effective foraging	F: Extended forelimbs; increase the surface area at the distal location for better assistance in orientational control during leaping and landing N: Irrelevant H: Hindlimb morphology and musculature that aids in running, jumping, and landing T: Increase surface area at the distal end of the long tail, which can be moved forward above the body or sideways with a fan-like feathered surface, for better assistance in controlling body axes during running	Y	Y	N	Y^b^	x	Y	Y
Hypothesis 4. Pouncing on prey^7^: It assumes that dinosaurs specialize in ambushing from elevated sites (e.g., trees; this element is shared with the “gliding” hypothesis) and in pouncing prey; the use of proto-wings and caudal plumage for drag-based control of their body orientation and trajectory during descent. Drag-based control is available to any animal with an aerodynamic surface, irrespective of whether that surface generates useful lift. Hence, even small proto-wings might be used to control the body orientation	Distal: no SD	Yes; no SD	No; higher crypticity is expected for effective foraging	F: Increase surface area at the distal location for better assistance in orientational control and a strong power stroke; the use of proto-wings for assistance in maintaining balance during the descent onto its prey N: Irrelevant H: Hindlimb morphology and musculature for climbing and balancing at elevated sites T: Tail with a distal surface for additional assistance in maintaining body balance when ambushing^42^ and descending from elevated places	Y	Y	N/x^c^	Y	x	N^d^	Y
Hypothesis 5. Insect net^5,6^: Use of distal proto-wings to capture escaping prey, similar to the way insect nets are used to catch prey by sweeping it against the prey	Distal; no SD	Irrelevant; no SD	No; higher crypticity is expected for effective foraging	F: Large and continuous trapping surfaces (feathered area) enhance insect net foraging efficiency; powerful ventral adductor muscles are needed to activate the large insect nets (feathered forelimbs). Long forelimbs with extended reach are essential for this function N: Long neck increases visual range by elevating the head, aiding in the efficiency of chasing prey. Precise visuomotor coordination enables accurate strikes to capture prey H: Hindlimb that facilitates fast running enhances the efficiency of insect net foraging T: long and stiff tail assists in motor control (balance and quick turns) during running	Y/N^e^	x	N	Y/N^e^	Y	Y	Y
Hypothesis 6. Immobilizing prey^9^: Use of proto-wings to maintain the body balance while grasping prey with the feet. Feathered forelimbs are used to restrict the prey’s escape route (‘‘mantling’’ the prey) while tearing the prey using jaws or a beak. The presence of sharp hook-shaped claws on hindlimbs has been proposed as an indicator of this type of prey handling, primarily in large predatory dinosaurs that hunt large prey. It is less likely to be observed in smaller theropods lacking such claws	Distal; no SD	Yes; no SD	Irrelevant	F: Increase surface area for better assistance in motor control (e.g., capable of generating relatively strong power stroke) during flapping; the powerful muscle, connected to the humerus, for sufficient stability in flapping N: Long neck enabling a dinosaur to reach down between its feet to handle prey held by the hindlimbs while also immobilizing it with the forelimbs H: Hindlimb anatomy for hooking and grasping prey, including hook-shaped claws for maintaining a grip on large prey T: Long feathered tail for better assistance in balance during stable flapping, aiding in the immobilization of prey	Y	Y	x	Y	Y^f^	N^g^	Y
Mechanisms not relevant to foraging: Hypothesis 7. Wing-assisted incline running^13,43,44^: Flapping proto-wings to create aerodynamic forces while running on inclined substrates	Distal; no SD	Yes; no SD	Irrelevant	F: Increase surface area for generating more vital aerodynamic forces during flapping. It involves a wide range in the pitch of the shoulders. The expected traits, such as the supracoracoideus muscle, do not align with the traits observed in basal pennaraptorans^4,45^ N: Irrelevant H: Hindlimbs for upward running, climbing, and balancing at elevated sites. However, even without special anatomical adaptations, upward running would still be possible. Therefore, the hypothesis remains feasible even in the absence of special hindlimb adaptations T: Tail with a distal surface that provides additional assistance in maintaining balance during climbing and aerial descent^44^	Y	Y	x	N	x	Y	Y
Hypothesis 8. Gliding^6,14^: It assumes that dinosaurs specialized in gliding from elevated sites (e.g., trees; this element is shared with the “pouncing *Proavis*” hypothesis). Use of surface area of proto-wings for gliding to reach particular destinations	Proximal; no SD	Yes; no SD	Irrelevant	F: Increase surface area at a proximal location for better gliding performance^7^ and better control and stability during gliding N: Irrelevant H: Hindlimb morphology and musculature for climbing and balancing at elevated sites, and hindlimb feathers for assistance in gliding T: Caudal plumage surface contributes to lift and stability during gliding^10,11,46^	N	Y	x	N	x	N	Y
HYPOTHESIS 9. Brooding^12^: Use of forelimb and tail feathers for nestling and chick-rearing, primarily involving the heating or shading of eggs and/or chicks	Distal; no SD	Yes; no SD	Irrelevant	F: The optimal posture (the extended forelimbs as in a brooding *Citipati* specimen^47^) to cover their nest N: Irrelevant H: The optimal posture (crouching like in a brooding *Citipati* specimen^47^) to incubate their eggs and/or to protect their chicks T: Tail feathers may provide additional assistance in brooding and/or shading their eggs and/or chicks	Y	Y	x	Y	x	Y	Y?^h^
HYPOTHESIS 10. Intraspecific displays^15,16,39,40^: Use of forelimb and tail feathers in visual displays; distinct color patch and/or ornament feather (e.g., elongated feather) are expected; high within-species differences (e.g., sexual dimorphism, ontogenetic variation) are expected	Distal; yes SD	Yes; yes SD	Present; yes SD	F: Increase surface area at distal and conspicuousness for a more vivid display; movements for assistance in displays N: Cranial ornamentations for a more vivid display H: Irrelevant T: Increase surface area and conspicuousness for a more vivid display; movements for assistance in displays	Y	Y	Y^i^	Y	x	x	Y

The flush-pursue hypothesis compared to previous hypotheses that may explain the function and evolutionary origin of proto-wings and caudal plumage in basal pennaraptoran dinosaurs. The predictions in this table are derived either directly from studies on avian flush-pursuers and their prey or from effects/mechanisms discussed in hypotheses proposed earlier in the literature.

^a^Although experiments with a robotic dinosaur based on *Caudipteryx* suggested a weak beneficial effect of proto-wings in running, as described in the “flapping proto-wings” hypothesis, it is uncertain whether such an effect was sufficiently strong in real *Caudipteryx*. Its anatomy suggests that the range of flapping movements might have been narrower than that of what was used in the robot.

^b^The basalmost pennaraptoran theropods possessed relatively small pennaceous surfaces on their forelimbs and tails. Therefore, the direct effect on the leap trajectory is expected to be small. However, the effect on body orientation (pitch and roll) during leaps is predicted to be sufficient to enhance the foraging efficiency, as evaluated in the theoretical calculations^8^.

^c^Color patterns are only known for a few species among the basalmost pennaraptoran theropods.

^d^Arboreal lizards have distinctly curved claws for climbing, a feather not observed in the basalmost pennaraptorans. Therefore, we assigned “N” for the prediction P4(H).

^e^The distal location of proto-wings is consistent with the “insect net” hypothesis, but short forelimbs are not consistent with it. Many pennaraptoran theropods and earlier dinosaurs seem to have relatively short forelimbs, especially in relation to their long necks, which can extend forward extensively during prey capture. This effectively precludes the use of short forelimbs as an efficient insect net. Additionally, the relatively limited range of forelimb movements, as reviewed in Methods Part 1–3, reduces the functionality of proto-wings as “insect nets”.

^f^The relatively long necks of basal pennaraptoran theropods would have allowed them to reach down to prey held by their feet. Their strong and hard beaks might have been easily used to handle and tear prey.

^g^Special hook-shaped claws are only applicable for large predatory dromaeosaurids, not for the basalmost pennaraptorans, which are the focus of our study. Therefore, we assigned “N” for prediction P4(H).

^h^As the tails are feathered, and multiple brooding specimens have been found only in pennaraptorans and not in non-pennaraptoran dinosaurs, we suggested “Y?” for prediction P4(T).

^i^Sexual dimorphism has not definitively been proven for any dinosaur due to small sample sizes and a lack of adult specimens. However, intraspecific signaling functions can also be performed by bright patches in monomorphic species.

## Results

We reviewed pennaraptoran dinosaurs (Methods part 1) and constructed a robot based on one of the basal species, *Caudipteryx* (Methods part 2). We then imitated avian flush-displays within the estimated motion range of *Caudipteryx* (Methods part 3) and examined the behavioral responses of grasshoppers (Methods part 4). The presence of distal proto-wings increased both the flush frequency and the distance at which the prey escaped (Fig. 2B; Table S1). Grasshoppers were flushed more frequently by the distally rather than proximally located proto-wings (Fig. 2C; Table S2). They escaped not only during the spreading stage of the flushing movement (Video S1, part 1) but also occasionally during the folding stage (Video S1, part 2). In additional separate tests conducted at a distance of 35 cm from the robot, 35% (7 out of 20) of grasshoppers escaped in response to displays that contained only the folding movements of the forelimbs with distal proto-wings. However, in response to the complete flush-display, comprising opening, pausing and folding the forelimbs with distal proto-wings, the majority of the grasshoppers (90%; 27 out of 30) escaped. This indicates that the opening stage of the display is crucial for achieving a high success rate in flushing the prey. Furthermore, grasshoppers escaped more often when white patches were present on the proto-wings (Fig. 2D; Table S3). The flushing rate was higher when caudal plumage was present on the tail (Fig. 2E; Table S4), particularly when the surface area of the caudal plumage was large (Fig. 2E; Table S4; Video S1, part 3).

In addition to the quantitative analyses of the behavioral experiments, we also measured the neurophysiological responses of LGMD/DCMD neurons (Fig. 1K; Methods part 5), known to be involved in jump-escape reactions in grasshoppers. These neurons were exposed to visual stimuli generated by animations that imitated the robot movements (Fig. 2F; Video S1, parts S4 and S5). The firing rate of the LGMD/DCMD pathway followed the pattern of angular speed during the opening and folding of the forelimb movements (Fig. 2G, Fig. S3A–D; Tables S5, S6). Two peaks, corresponding to the opening and closing forelimb motions, were observed in the firing rate (Fig. 2G; Fig. S3B–D). The size of these two peaks was higher when proto-wings were present than when they were absent (Fig. 2G; Fig. S3B–D; Table S7). These results align with the results of our behavioral experiments.

## Discussion

We propose that the flush-pursue hypothesis provides a new perspective on the evolution of pennaceous feathers. This hypothesis suggests that the exploitation of prey escape behaviors, based on the concept of a “rare-enemy”, might have played a role in the evolution of early pennaceous feathers. Hence, the premise of the flush-pursue hypothesis is that this behavior evolved among early pennaraptorans but not among all other predators occupying a similar dietary adaptive zone. To assess the flush-pursue hypothesis, we used a *Caudipteryx* robot and animations to examine the behavioral and neurophysiological responses of grasshoppers to hypothetical dinosaurian flush displays. The results from both behavior and neurophysiology align with existing literature on visually evoked jumps in Orthoptera^41,48,49^, supporting the idea that proto-wings, especially those located distally and with contrasting patterns, as well as feathered tails in predatory dinosaurs, might have been used to exploit their prey’s escape responses, similar to how avian flush-pursuers exploit insect escape reactions.

The comprehensive nature of the flush-pursue hypothesis is apparent when compared with the other hypotheses. Its capacity to explain various aspects is summarized in Table 1. The flush-pursue hypothesis can account for not only the presence, distal location, and color contrast in the pennaceaous plumage on the limbs and/or tails of pennaraptoran dinosaurs but also for a set of other morphological features. These features encompass a relatively small body, as well as morphological adaptations for fast cursorial locomotion, maneuverability, quick forward attacks on prey, and good visuo-motor control (Table 1).

The flush-pursue hypothesis is consistent with the presence of pennaceous proto-wings in relatively small theropods^50^ who hunted arthropod prey^31^. This is the case even though feathered body covering was common across a wide range of body sizes, including large carnivores^51^. The small body size of arthropod-hunting theropods equipped with proto-wings is consistent with, or at least not contradicted by, some of the foraging-related mechanisms previously proposed (e.g., hypotheses 2–4 in Table 1: the “flapping proto-wing”, “leaping”, and “pouncing on prey” hypotheses). This is because the aerodynamic advantages related to lift or drag generated by the surfaces of pennaceous feathers, as suggested in those hypotheses, are more pronounced in small theropods^8^. These mechanisms are incorporated into the comprehensive flush-pursue hypothesis (Table 1). Therefore, the flush-pursue hypothesis also appears more aerodynamically feasible in small theropods, facilitating rapid changes in pursuit speed and direction (Table 1).

The concept of body miniaturization^50^ and the “insect net” hypothesis (Hypothesis 5 in Table 1), which is also incorporated into the flush-pursue hypothesis, are linked to a shift in diet towards arthropod prey (including pure insectivory as well as omniovory, as observed in the extant avian flush pursuers). This dietary shift is also consistent with the idea that flush-pursue foraging occurred in relatively small pennaraptoran dinosaurs: the flush-pursue strategy is particularly efficient when the prey possesses a visual and neural system that struggles to accurately assess distance to predator, predator speed, size, or type^20^. This highlights the critical role of simple looming-sensitive circuits in triggering escape responses in prey animals. The relative simplicity of these circuits is found in arthropods, such as insects^25,26^ or crabs^27,28^, which are susceptible to exploitation by avian flush-pursuers. This explains why the flush-pursue strategy is primarily observed in insectivorous or omnivorous birds but not among purely carnivorous ones (Fig. S1A; Text S1; carnivory defined as including vertebrates in the diet). Therefore, within the framework of the flush-pursue hypothesis, the proto-wings are expected to have evolved in the smaller insectivorous (or omnivorous) rather than in the larger carnivorous theropods whose prey is more likely to have sensory and cognitive abilities that allow them to estimate predator distance and type accurately. Consequently, they can respond more specifically to different situations and predator types.

The hypothetical flush-pursuing pennaraptorans could have potentially benefited from the morphological evolution in their endocranium^52^ and inner ear^53^ features observed in their fossils. These adaptations might have aided in precise control during fast pursuits, such as running and jumping to capture prey. Hence, the proposed presence of flush-pursue foraging among basal pennaraptorans might have contributed to the observed evolutionary trends in the endocranium and inner ear that resulted in an intermediate morphology between bipedal non-pennaraptoran and avialan dinosaurs^52,53^. These morphological features are not consistent with, nor essential for, some alternative hypotheses, such as those related to socio-sexual displays or brooding behaviors. These alternatives are not incorporated in the comprehensive flush-pursue hypothesis (Table 1).

The flush-pursue hypothesis can be applied to other bipedal vertebrates with a wide forelimb surface area. Our study shows that any increase in the visually perceived surface area of forelimbs, whether through the growth of pennaceous or non-pennaceous feathers, pro- and post-patagia, or membranes, helps in flushing prey for any cursorial hunter that pursues and captures its prey. Some key features similar to pennaraptoran dinosaurs, such as bipedalism and miniaturization in scansoriopterygid theropod dinosaurs and the precursors of the Pterosauromorpha^54^, suggest the potential involvement of fast bipedal pursuits after prey. However, due to a large gap in the fossil record, the hypothetical intermediate stages of development of forelimb surfaces are unknown. Consequently, the testing of the role of flush-pursue foraging in the initial expansion of their forelimb surfaces is hindered by the lack of fossil evidence.

We also consider the possibility that colors and feathery surfaces might have initially evolved for non-foraging purposes, as suggested by some existing hypotheses (Table 1). Subsequently, they could have been used in flush-pursue foraging. This potential adaptation would have exposed these features to natural selection pressures, leading to the co-evolutionary reinforcement of adaptations that serve the main functions (flushing, pursuing, and capturing/handling the prey) associated with flush-pursue foraging. Furthermore, these adaptations could have laid the foundation for the subsequent evolution of wings and powered flight. Therefore, we propose that the comprehensive flush-pursue hypothesis does not contradict many existing hypotheses, especially those related to foraging. Instead, it provides a common ground for combining these hypotheses into a network of interrelated mechanisms, each mutually reinforcing the other. This perspective emphasizes the complexity of feather origins and evolution, acknowledging that multiple factors may have contributed to the development of ‘proto-wing’ structure and color. In this broader context, we highlight the significance of flush-pursue behavior as a valuable addition to the ongoing discussion on the early evolution of ‘proto-wing’.

## Conclusions

The flush-pursue hypothesis offers a new comprehensive perspective on the evolution of pennaceous feathers in non-avian dinosaurs. Based on our empirical results and comparisons with previously proposed hypotheses, we argue that flush-pursue foraging in a “rare-enemy” context might have played an essential role in the evolutionary processes that shaped several interrelated functions of proto-wings, feathered tails, and other aspects of dinosaur morphology. Our results emphasize the significance of considering sensory aspects of predator–prey interactions in the studies of major evolutionary innovations among predatory species.

## Methods

### Part 1. *Caudipteryx* as a model for building a robot

The decision to use *Caudipteryx* as the model for building a robot was based on the following information. Pennaceous feathers with a rachis structure are restricted only to Pennaraptora (Oviraptorosauria + Paraves^2^), indicating that the evolution of wings for aerial locomotion occurred within this clade. Oviraptorosauria is the most phylogenetically basal clade in Pennaraptora. We selected the forelimbs of the oviraptorosaur *Caudipteryx* to represent the ancestral condition of Pennaraptora because *Caudipteryx* is one of the basal taxa with almost completely preserved Oviraptosaur with ‘proto-wings’^55^. We examined the following *Caudipteryx* specimens described in the literature: *C. zoui* [NGMC 97-4-A (holotype) and NGMC 97-9-A (paratype) of Ji et al.^3^; BPM 0001 of Zhou et al.^56^; PMOL AD00020 of Li et al.^57^], *C. dongi* [IVPP V 12344 (holotype) of Zhou and Wang^58^], *C.* sp. [IVPP V 12430 of Zhou et al.^56^; LPM0005 of Feduccia and Czerkas^59^].

Pennaceous feathers (open-vaned, broad, and ‘frond’ shaped) on the forelimbs and tail of *Caudipteryx* are symmetrical and highly simplified compared to those of flying birds, both extant and extinct^3,60,61^. The pennaceous forelimb feathers of *Caudipteryx* are located distally, and the pennaceous tail feathers are restricted to the tail tip^3,55,61,62^. Although the tail feathers are pennaceous (vaned), no specimen preserves evidence of hooklets on the barbules^3^. In *Caudipteryx* (IVPP V22606), two layers of tail feathers are evident: one layer of shorter rachis-less body feathers and another layer of longer pennaceous tail feathers^63^.

The feathered forelimbs of *Caudipteryx* could have produced weak aerodynamic forces during rapid terrestrial locomotion^64^. However, feathers on the forelimbs and tail of *Caudipteryx* are likely unrelated to flight because they have no striking aerodynamic features and no osteological features that would support any aerial capability^60^. Other functions, such as maintaining balance or producing additional thrust during running or climbing, insulating eggs, and displaying, are all viable hypotheses^60^. *Caudipteryx*, with its center of mass positioned anteriorly, probably used a running mechanism different from the more basal bipedal dinosaurs^65^ and more similar to that of modern cursorial birds, some of which are flush-pursuers (see Text S1).

The evolution of a predator’s plumage that visually contrasts with the background appeared to be advantageous in flush-pursuit foraging^66^, and light patches contrasting with the darker plumage on the wings and tails of birds were also shown to be advantageous in the context of flush-pursue foraging^66^. Considering that both the tail and body feathers of *Caudipteryx* are known to be black^57^, and visible banding patterns are shown in the tail feathers (striped caudal plumage^3,67^), *Caudipteryx* is a reasonable model species for evaluating the effect of the color pattern of proto-wings/caudal plumage on flushing performance in non-avian dinosaurs.

The decision to use *Caudipteryx* as the model for building a robot should be viewed in light of the following remarks:

1. *Caudipteryx* is from the early Cretaceous (Barremian-Aptian), whereas the first pennaraptorans are thought to have emerged as late as the middle Jurassic, as evidenced by the more derived *Archeopteryx*^68^. However, to infer the ancestral state of the most basal pennaraptorans, using basal taxa rather than the oldest taxa is likely more reliable, pending the discovery of Jurassic caudipterids.
2. The preservation of gastroliths in several *Caudipteryx* fossils^3,69^ indicates that the diet might have included hard plant materials^31^. However, gastroliths may also indicate a diet of arthropods with hard exoskeletons (suggested in extant lizards^70,71^), suggesting an omnivorous diet. In general, the most basal Pennaraptora, Oviraptorosauria, and the derived Paravian theropods, Deinonychosauria, showed a diversity of feeding ecology, including carnivory, insectivory, omnivory, and herbivory^31^, a situation similar to the extant avian flush-pursuers^72–74^, for which the flush-pursue strategy is one of several tactics employed during foraging.
3. *Caudipteryx* shows particularly short arms and tails^16,75^ with a reduced third finger, all of which are derived condition in Oviraptorosauria (the most basal clade of pennaraptoran dinosaurs^16^). Thus, if the experimentally imitated flush-displays by the relatively short-armed robot generally similar to *Caudipteryx*^16,75^ or *Incisivosaurus*^1,76^ will prove efficient in flushing arthropods, then it is likely that this function will be even more pronounced in other basal pennaraptoran with longer forelimbs, such as *Protarchaeopteryx*^3^ and Scansoriopterygids^77^.
4. The arms of many small-bodied theropod dinosaurs, even those believed to have predatory habits, were relatively short and might not have been used in the capture stage. Instead, it is suggested that they used their long and robust necks for quick forward movements to capture prey efficiently^78^. However, the forelimbs, equipped with claws, could have been used to handle the prey after capture.
5. *Caudipteryx* shows some disparity in feather size and distribution among caudipterids: pennaceous feathers are more restricted to the distal portion of the forelimb (with shorter secondary feathers) and tail in *Caudipteryx* than in other members of Caudipteridae [e.g., *Incisivosaurus*^1,76^ and *Xingtianosaurus*^79^]. However, since *Caudipteryx* specimens offer the most complete and comprehensive data among caudipterids and possess a relatively distal distribution of pennaceous feathers on the forelimbs and tail (which would likely enhance efficiency in flush-pursuit foraging), we think that using *Caudipteryx* as a model for the ancestral early-diverging pennaraptoran is both conservative and based on the best available data.

In conclusion, *Caudipteryx* still serves (since its initial report in 1998) as the most representative basal-most pennaraptoran in the fossil record currently known, and we aim to shed light on the evolution of proto-wings and caudal plumage considering the following assumptions:

1. early members of Pennaraptora generally possessed similar proto-wings’ dimensions (relative to body size) to those of *Caudipteryx*;
2. their forelimb movement range was anatomically restricted in a manner similar to *Caudipteryx*;
3. early members of Pennaraptora hunted small prey such as insects, crustaceans (e.g., crabs), small reptiles, and small mammals that used visually triggered escape behaviors to avoid predation;
4. early members of Pennaraptora were skilled in chasing (running) and capturing flushed prey.

### Part 2. Building a robot

Based on the skeletal and plumage anatomy of fossil specimens of *Caudipteryx*, we built a robot (Robopteryx; Figs. 1H, 3, Fig. S2) of a size similar to that of *Caudipteryx*. Of the known *Caudipteryx* specimens, we chose IVPP 12430^56^ for the overall body proportions (length of body, hip height, length of arms, and tail), IVPP 12344^58^ for the shape of the proto-wing, and NGMC 97-4-A for the tail feather dimensions and pattern^3^, and PMOL AD00020 for coloration^57^. The dimensions of arms and proto-wing referred to the identical specimens as in Talori et al.^10^. There is an indication that the presence of the propatagium should be treated tentatively^51^. However, based on the visible contour of what is presumed to be the propatagium and as inferred from the preserved positions of the forelimbs, we imitated the propatagium based on LPM0005^59^.

As the tail feathers of reported *Caudipteryx* specimens are folded in half, the opened outline was inferred from the tail fan of *Incisivosaurus* STM22-6^1,76^. We took a conservative approach, assuming that the folded tail feathers of PMOL AD00020 represented the anteriormost margin of the opened tail fan and that the distal-most feathers would have filled in the gap of the fan, as in STM22-6, forming a continuous fan.

The robot (Fig. 3) was built from aluminum (A6061; the CAD used to build the robot is shown in Fig. S2A,B). Proto-wings and caudal plumage were made from black-colored paper (Fig. 3C,D), with plastic pieces inserted between segments of the proto-wing (arrows in Fig. 3D). We used black elastic stocking (Fig. 3B) to imitate the propatagium, and the head was built using black-colored polystyrene (Figs. 1H, 3A). To imitate a bent tail, an additional structure was attached to the tail part (Fig. 3C). The main body was covered with black-colored felt. The robot’s forelimb and tail motions were driven by a tendon-driven mechanism controlled by custom-made software running on a mobile phone (see details in Fig. S2).

### Part 3. Choosing robot postures and movements

We determined the hypothetical resting posture angles and motion ranges for *Caudipteryx* based on relevant literature.

We considered five angle types (Fig. 3G,H). Four of these angles are defined in the side view of the robot (Fig. 3G, upper row; Fig. 3H):

- Shoulder angle (S)—The angle between the humerus structure and a horizontal line parallel to the lower part of the main body.
- Elbow angle (E)—The joint angle at the elbow.
- Wrist angle (W)—The joint angle at the wrist.
- Tail angle (T)—The angle between the tail structure and the main body.

One angle is defined in the frontal view (Fig. 3G, lower row):

Lift angle (L)—the angle between the humerus structure and the vertical line running along the side of the main body.

#### Forelimb resting posture

Based on the literature^80^, the angles that imitate the resting posture of *Caudipteryx* should be set as follows: ~ 33° for S, ~ 106° for E, and ~ 131° for W. However, due to a design limitation of the robot (one motor controlling both elbow and wrist joints), we set the values of E and W to be identical: ~ 106°. This setting allows the robot to mimic the spreading and folding of the arm as a consequence of automatic wrist folding^81^, which has been observed in volant birds^81^ and, more recently, in alligators and ostriches^82^. Therefore, by using the extant phylogenetic bracketing approach^83^ and considering the presence of propatagium^59^, we can reasonably expect *Caudipteryx* to have used a similar mechanism, as has been proposed even for *Chilesaurus*^84^ of debated affinity inside Dinosauria^85–87^.

In summary, we set the following values for the resting posture: S = 33°, E = 106°, and W = 106° (Fig. 3G). We set the resting angle L as 12°, a consequence of the robot’s forelimb structure (Fig. 3G).

#### Forelimb motion

Estimating the range of motion from anatomy helps to infer joint mobility in real animals in vivo^82^. However, determining the exact range of motion from *Caudipteryx* bones is challenging due to the compression of bones during fossilization processes in known specimens. To address this, we used a conservative method of phylogenetic bracketing. This method involved selecting model organisms that represent both more basal (*Acrocanthosaurus*) and more derived (*Bambiraptor*^88^) conditions, and then assuming that the range of motion at the shoulder joint of *Caudipteryx* fell between these two species. In other words, the data for *Acrocanthosaurus* and *Bambiraptor* provided estimates for the minimum and maximum range of motion (Table S8), respectively, in the phylogenetically intermediate *Caudipteryx*. Morphology of the articular surface of the glenoid, where the upper arm (humerus) meets the shoulder (scapula) in the *Caudipteryx* specimens, indicates that they were unlikely to have been held over horizontally (Senter^88^, *contra* Talori et al.^10^), or that they have had a range of motion seen in more derived dromaeosaurids, much less than that seen in birds. The range of shoulder-raising motion may have been closer to *Acrocanthosaurus*^89^, in which the arm could not be raised to a horizontal position^90^. The elbow flexion is beyond 90 degrees in Ornithomimosauria and more derived clades^91^. We suspect that the range of motion in the elbow might be intermediate between that of *Acrocanthosaurus* and *Bambiraptor*, with folding movements closer to *Bambiraptor*^90^. As for the wrist, the radial angles of *Caudipteryx* imply a greater range of abduction than observed in dromaeosaurids^92^. Therefore, we assumed that the wrist could fold like some extant birds but could not be held completely straight^92^. In conclusion, we hypothesized that *Caudipteryx* might have used the following approximate ranges of the four angles (Fig. 3G,H; Table S8): (− 19° ~ 2°) ≤ S ≤ (114° ~ 123°), 55° ≤ E ≤ 136°, 0° ≤ W < 180°, L ≤ 88°.

Finally, we selected the hypothetical forelimb flushing movements within the estimated motion range (Video S1, part 6). The robot’s forelimbs were lifted from the resting posture (S = 33°, E = 106°, W = 106°, L = 12°) to the estimated maximum value for each angle (S = 123°, E = 136°, W = 136°, L = 88°). This lifting process took 0.42 s, followed by a 0.2 s pause. Subsequently, the forelimbs were reverted back to the resting posture, and this closing process also took 0.42 s. This robot’s forelimb movements resemble the wing movements of the ground-foraging avian flush-pursuers [e.g., Greater Roadrunner (*Geococcyx californianus*; link 1 provided in Text S1), Rufous-tailed Scrub Robin (*Cercotrichas galactotes*; links 21 provided in Text S1)] albeit the Greater Roadrunner’s flushing movement takes shorter duration of about 0.23 s for wing spreading and folding. Technical constraints in the robot’s design did not allow for faster movement. The movements of the robot’s forelimbs are shown in Video S1, parts 1, 2, and 5.

#### Tail motion

With proportionately well-developed tail muscles such as *Musculus longissimus* and *Musculus ilio-ischiocaudalis*^16^, oviraptorosaurs such as *Caudipteryx* would have been capable of swinging and twisting their tails both mediolaterally and dorsoventrally with a degree of muscular dexterity beyond that of most other theropods and modern reptiles^16^. However, in the robot, we only used a simple vertical up-down movement imitating the tail-flushing movements of some of the extant flush-pursuers (Text S1): the value of T during tail movement changes from 150° to 90° (this process takes 0.33 s), and then reverts to 150° (this process takes 0.33 s; Video S1, part 3). Sidewise movements with the tail, present in some flush-pursuers, were impossible due to the robot’s design constraints.

### Part 4. Behavioral experiments

#### Study site and study species

We conducted behavioral experiments on the band-winged grasshopper *Oedaleus infernalis* (Orthoptera), which can serve as a model prey susceptible to flush-pursue foraging. Species identification was made using field guides^93,94^. As the escape behavior of orthopterans is likely affected by sex^95^, we tested adult males only, which are easily identifiable based on the body shape and size without capturing the animals.

Orthoptera is an ancient prey taxon^96^ whose members use a fast escape reaction as an adaptation to evade attacking predators^96^. Grasshoppers may be unable to precisely evaluate the distance, size, and type of an attacking predator due to constraints of their sensory systems, including relatively poor visual resolution and close distance between the eyes. They use relatively simple looming-sensitive neural circuits that mediate visually triggered escapes in response to looming objects^49,97^, including fast-approaching predators.

From August to September 2020 and 2021, we conducted behavioral experiments on males *Oedaleus infernalis* (Fig. 1J) along a 2-km-long trail (37° 40′ 12.3″ N, 126° 53′ 11.4″ E) in Go-yang and a 1-km-long trail (35° 42′ 00.2″ N 128° 27′ 29.0″ E) in Dae-gu, South Korea. We chose grasshoppers resting on the road/path where the robot could be easily placed facing the grasshopper without much disturbance. We tested the grasshopper’s escape frequency in response to the robot’s movements (see the experimental treatment description below).

#### General experimental design

Due to technical constraints, we were unable to fully imitate the natural body movements involved in foraging activities. As a result, we decided to focus on imitating specific moments in the flush display observed in some flush-pursuing birds. During these moments, the bird remains stationary, and the major visual cues for nearby prey are the movements of their wing and/or tail. Our experiments followed a general procedure (e.g., Fig. 1H): (1) gently place a 1 m-long wooden stick with scale marks next to the grasshopper, (2) take a picture of the grasshopper and record its body orientation relative to the robot’s position, (3) place the robot at a specific distance from the grasshopper, depending on the experiment, (4) run the robot’s forelimb or tail movements using a phone wireless controller software, (5) if the grasshopper escapes at the first (the farthest) distance, the experiment on the individual is over, (6) if the grasshopper does not respond to the robot’s movements, carefully and slowly move the robot to the following test distance closer to the grasshopper and repeat (4), (5), (6) until the grasshopper responds or until the closest distance to the grasshopper planned in the experimental design is reached. The slow and careful placing of the stick and the robot might have already drawn the grasshopper’s attention to the robot in a way that is probably similar to the stimuli perceived by prey from the walking by the ground-foraging flush-pursuers. These birds walk short distances in-between delivering their flush displays (e.g., greater roadrunners, northern mockingbirds, striped cuckoos, or rufous-tailed scrub robins; see Text S1).

To place the robot in front of the grasshopper, we carefully moved the robot using a long beam that was attached to the robot. We tested 3 to 5 individuals in one experimental treatment, followed by 3–5 tests in the subsequent treatment (and in some experiments followed by a third experimental treatment on 3–5 individuals), after which we returned to using the first treatment. We repeated this cycle for several hours daily, resulting in no bias among treatments regarding the time of day. To avoid any potential effects of shadows created by the robot's movements on the grasshopper’s response, we placed the robot in positions where no shadows would appear near the grasshopper while the robot’s forelimbs or tail were in motion. The main body of the robot was tilted 37° upward for experiments concerning proto-wings (experiments 1–3; Fig. 3E) to imitate a posture observed in ground-foraging flush-pursuers that use wing displays, such as greater roadrunners, northern mockingbirds, or rufous-tailed scrub robins (see Text S1). In the case of the experiment concerning caudal plumage (experiment 4; Fig. 3F), the main body of the robot was tilted 40° downward to imitate a situation where the body is tilted forward during tail displays, similar to the body pivoting observed in some flush-pursuers like *Myiobrous* redstarts with upward-lifted and spread tails or similar to the foraging movements of the willie wagtail (*Rhipidura leucophrys*) when the tail is quickly cocked upwards while the head points downward.

#### Experiment 1. The effect of proto-wings and motor sound on flushing performance.

We used three experimental conditions (Fig. 2A): (1–1) robot presented without forelimb movements but with the sound of the robot played back through a speaker; (1–2) movements of forelimbs without proto-wings; (1–3) movements of forelimbs with distal proto-wings. Since auditory cues are also used to detect predators^95^, condition (1–1) was used to determine the effect of noise generated while the robot operates. The motor noise was recorded using a microphone (BY-MM1, BOYA) connected to a smartphone before the experiments, and it was played through a speaker (XMYX03YM, Xiaomi) attached to a structure between the robot’s legs at an amplitude similar to that of the robot’s operational noise. The test distances were 100, 80, 60, 40, and 20 cm (between the grasshopper and the point between the robot wheels). We found that the motor sound rarely affected the behavior of grasshoppers (only two individuals jumped away out of a total of 46 tests in the 1–1 condition) and that the remaining treatments with moving forelimbs triggered escapes significantly more often than the sound-only treatment: Dunn’s test with Bonferroni correction, *P* for (1–1) vs. (1–2) < 0.001, (1–1) vs. (1–3) < 0.0001 (Table S1). Given these comparisons, we regarded that the effect of the sound on grasshoppers’ escape behavior was negligible. Consequently, we focused on comparing between the two remaining treatments (i.e., 1–2 vs. 1–3), which are presented in the main text Fig. 2B.

#### Experiment 2. The effect of the presence and location of proto-wings on flushing performance.

For efficient gliding, development of surfaces near the body is expected^7^. However, for efficient flushing and pursuing prey, development of surfaces on the distal parts of the forelimbs is expected because it produces a relatively stronger visual stimulus during limb movements. Therefore, the flush-pursue hypothesis predicts that proto-wings are located proximally to maximize flush performance. To determine the effect of the presence and location of the proto-wings on flushing performance, we tested grasshoppers in three experimental treatments (Fig. 2A): (2–1) proto-wings absent; (2–2) proximal proto-wings present; (2–3), and distal proto-wings present. Proximal and distal proto-wings have an identical surface area (128 cm^2^) to the distal proto-wings at the peak of the visual stimulus of the flushing movement (right before folding the forelimbs). Based on the results of Experiment 1, we chose 70 and 35 cm as test distances to simplify the experimental procedure in Experiment 2. None of the grasshoppers responded to the robot’s flushing movement at 70 cm. Therefore, we only used the responses at 35 cm for statistical comparisons among the treatments. We also noticed that the grasshoppers escaped during both the forelimbs’ spreading stage and the folding stage of the flushing movement. We thus conducted an additional experiment to compare the effect of opening-only vs. folding-only movement on the grasshoppers’ escapes at 35 cm.

#### Experiment 3. The effect of proto-wings’ color contrast on flushing performance

To determine the effect of proto-wings’ color contrast on flushing performance, we tested grasshoppers in two experimental treatments (Fig. 2A): (3–1) plain black proto-wings; (3–2) white-patched proto-wings, created by applying white paint to create a hypothetical stripe pattern on the black proto-wings. As none of the grasshoppers escaped at a distance of 70 cm in Experiment 2, we chose shorter test distances: 60 and 50 cm. Also, considering that 90% of the grasshoppers escaped in response to the robot’s flushing movement equipped with proto-wings in Experiment 2 at a distance of 35 cm, we chose a slightly greater distance of 40 cm as the nearest test distance so that we can observe differences between the plain black proto-wings and the white-patched proto-wings treatments in terms of escape frequency. Hence, in Experiment 3, we used three subsequent distances in the field procedure: 60, 50, and 40 cm. As the grasshoppers escaped only at the 40 cm distance, the statistical comparison between the two treatments was conducted only on the results obtained from the 40 cm tests.

#### Experiment 4. The effect of the presence and area of caudal plumage on flushing performance

We tested grasshoppers in three experimental treatments (Fig. 2A): (4–1) caudal plumage absent; (4–2) normal-sized caudal plumage present (262 cm^2^); (4–3) large-sized caudal plumage present [twice the surface area of (4–2), 524 cm^2^]. The effect of upward tail movements (the only type that our robot could imitate) can only be expected when the tail view is not blocked by the head, neck, and body of the robot. Therefore, we considered a hypothetical situation in which the predator already focuses on the ground with its head slightly down and body tilted downward (Fig. 3F). We conducted our tests at two distances from the grasshopper: 80 and 60 cm, resulting in much closer distances to the immobile, downward-tilted head. Specifically, the distance between the head and the grasshopper was 10 and 30 cm, respectively.

#### Analysis

All statistical analyses were conducted in R version 4.0.3^98^. The Dunn’s test with Bonferroni correction was used to determine differences in flushing performance through multiple pairwise comparisons using “dunn.test” function from the dunn.test package^99^ for Experiments 1 and 4. For Experiments 1 and 4, we considered the distance at which the grasshopper escaped [e.g., 100, 80, 60, 40, 20, 0 (= “no response”) in Experiment 1] as the dependent variable. The experimental treatment was used as the independent variable.

In Experiment 2, the Chi-square test with Bonferroni correction was performed to determine differences in flushing frequency between the experimental treatments using the “pairwiseNominalIndependence” function from the rcompanion package^100^. In Experiment 3, the Chi-square test with Yates’ continuity correction was performed to determine differences in flushing performance among experimental treatments using the “chisq.test” function from the stats package^98^. For Experiments 2 and 3, the grasshopper’s escape behavior (binary variable: escaped or not) was used as the dependent variable, with the experimental treatment as the independent variable.

Occasionally, the grasshopper escaped while the robot was being moved between distances before the robot’s movement was displayed. These data were excluded from the statistical analyses. Given that we repeatedly switched experimental conditions throughout the day, the potential effect of temperature was not addressed in the statistical analyses. Additionally, we could not control the grasshopper’s body orientation relative to the robot’s position during the experiment. The pattern of escape neurons’ firing activity may vary depending on the eye region facing the display^101^, and a frontal approach of a visual stimulus shows a different escape pattern compared to other approach directions [from the side and back^102^]. Therefore, we conducted two statistical analyses: one using the entire dataset and another using a smaller dataset after removing data points from tests when the robot was placed in front of or behind the grasshopper. Both sets of analyses led to the same conclusions. The statistical significance of the effects tested in the experiments is presented in the figures as asterisks, and more detailed information is given in Tables S1–S4.

### Part 5. Extracellular neurophysiological recordings from the grasshopper’s LGMD/DCMD pathway

#### Animations

This experiment aimed to illustrate the increased intensity of the grasshopper DCMD neuron’s response to a specific display movement type, known to be used by extant flush-pursuing birds and imitated in our behavioral experiments. A comprehensive study will evaluate the full spectrum of wing and tail displays observed in avian flush-pursuers.

Similar to the displays presented by the *Caudipteryx* robot (Robopteryx) in the behavioral experiments, we created *Caudipteryx* animations based on the morphology and size of *Caudipteryx* specimens (see Methods part 2 for specimen information) using a 3D animation software: Blender (version 3.2.0). The animations imitate the dinosaur, similar to the robot, with the exception of the neck and head, which are represented in a more naturalistic manner in the animations.

In these animations, as with the Robopteryx, the hypothetical dinosaurian flush-pursuer moves its forelimbs from the estimated resting posture (S = 33°, E = 106°, W = 106°, L = 4°) to the estimated maximum value of each angle (S = 123°, E = 136°, W = 178°, L = 88°; this process takes 0.23 s), followed by a 0.1 s pause, and then reverts to the forelimbs’ resting posture along the same trajectory as the expansion trajectory (this process takes 0.23 s; see Methods part 3 for the estimated motion range information). We produced two animations of the forelimb-flushing dinosaur: one without and one with distal proto-wings (Video S1, parts S4 and S5).

Additionally, we created an animation of a simple looming stimulus (an approaching circle; *l*/|*v*|= 5 ms, where *l* is the radius of 3 cm and *v* is the imitated constant approaching speed of 6 m/s; Video S1, part 7) similar to the classical stimuli that have been used for over 45 years in classical neurophysiological studies of the LGMD/DCMD pathway^41,48,103–105^.

In the animations, both the dinosaur and the circle are depicted in black (R—000, G—000, B—000), and the background is colored light gray (R—203, G—203, B—203). Since we placed the grasshopper ventral side up in the experiments, the hypothetical dinosaurian flush-pursuer was oriented upside down in the animations (Fig. S4B).

#### Study subject

We used adult males of the band-winged grasshopper, *Oedaleus infernalis*, collected from the study sites where the behavioral experiments were conducted. We kept them in an indoor breeding facility and provided them with grass for food.

#### Laboratory set-up

We used tape to fix a grasshopper onto a corkboard ventral side upward (Fig. S4A–C). We removed the antennae to prevent noise and prevent accidental obstruction of the view. Then, we slightly tilted the head backward using a pin to expose the neck connectives (between the head and thorax). Beeswax was added to both sides of the neck to keep the saline solution in there. One eye (the left one) was covered with beeswax to block the view. Next, we carefully dissected the soft ventral part of the neck to expose the ventral nerve cords (Fig. S4D). We dropped the saline solution (NaCl 210 mM, KCl 7.1 mM, CaCl_2_ 9.0 mM, Tris-buffered to pH 6.8) on the part and hooked an extracellular silver wire electrode (127-µm bare diameter, AM systems) to the contralateral nerve cord (Fig. S4E,F). The other wire of the electrode with a pin is pinned on the abdomen (Fig. S4C).

We used a stereoscope during the dissecting and placing the electrode. The electrode was attached to an electrode holder (H-13, Narishige), and the holder was manipulated using a Micromanipulator (MM-3, Narishige). The electrode was connected to the Neuron SpikerBox Pro (Backyard Brains, USA), which was connected to a laptop. The BYB Spike recorder (Backyard Brains, USA) was used on the laptop to record neural activity in response to *Caudipteryx* animations at a sampling rate of 10 kHz. During the recording from the nerve cord, the BYB Spike recorder displayed DCMD spikes in real-time. The animations were projected on a flat-screen monitor (TFG32Q14P IPS QHD 144, Hansung computer; 32 Inch) with a display brightness of 400 cd/m^2^ and a refresh rate of 120 Hz. The distance between the monitor and the grasshopper was set to 35 cm (Fig. S4A). To reduce noise in the recordings, we used a separate cable to connect the Neuron SpikerBox Pro, Laptop, and Micromanipulator to the ground. To synchronize neural activity and visual stimuli, we used an iPhone 12 mini to record high-speed video (240 fps) of both the animation screen and the screen displaying neural spiking in real-time.

We followed numerous previous studies, as exemplified by several classical studies^41,48,103–105^, in identifying DCMD spikes in the recordings. The DCMD spike sorting was based on spike amplitude, general shape, and response pattern. We confirmed that the black looming circle stimulus (*l*/|*v*|= 5 ms) displayed on the monitor triggered the grasshopper’s typical spiking frequency response to a fast looming stimulus (Fig. S3H, Table S9), characterized by an accelerating increase in firing rate up to the maximum point, followed by decreases.

#### Experimental design

To determine the effect of the presence of proto-wings on the neural response of the LGMD/DCMD pathway, we compared the responses of grasshoppers to two forelimb animations: (1) display without proto-wings (NoPW treatment); (2) display with distal proto-wings (PW). Each animation was played six times to each grasshopper, following one of the two experimental orders: (PW, NoPW, PW, NoPW, PW, NoPW, PW, NoPW, PW, NoPW, PW, NoPW) or (NoPW, PW, NoPW, PW, NoPW, PW, NoPW, PW, NoPW, PW, NoPW, PW). Additionally, for each individual, we played a looming circle animation (black circle of *l*/|*v*|= 5 ms) at the beginning and at the end of the recording session. A 1 min pause followed each stimulus presentation. Hence, the duration of a set of experiments for each individual was approximately 20 min.

#### Analysis

We analyzed the neural spike data using Spike2 software (version 5, Cambridge Electronic Design, Cambridge). First, we inspected the recorded firing rate with a bin size of 10 ms (Fig. S3I), and we realized that even at this relatively narrow bin size, the maximum spiking frequency is consistently observed at the beginning of the display (within the first 10 ms). To analyze the differences in the firing rate profile between the two treatments, we used a wider bin size (25 ms). This broader bin size produces a more general view of the response, better suited for our comparisons, and has been previously used in classical neurophysiological studies of the LGMD/DCMD pathway [e.g.,^103^].

We used a linear model, using the “lm” function from the stats package^98^, to determine the effect of the presence of proto-wings on the peak size of the DCMD firing rate. The dependent variables in our model were the two peaks generated during the response to the opening and folding forelimb motions. We used two fixed effects: treatment (proto-wings presence vs. absence) and individual ID (with three levels). The normality of the model residuals was checked using the Shapiro–Wilk test^98^.

For each frame of the looming circle animation, we determined the angular size subtended by the circle on the retina and calculated the changes in the angular speed of expansion during the animation. In the case of the forelimb animations, we determined the angular distance between the tips of the left and right forelimb (i.e., wing span; proto-wing tips were used for the “with proto-wings” animation). We used this information to calculate the angular speed based on the changes in the angular wing span.

### Supplementary Information

Supplementary Information.

Supplementary Video 1.

## Data Availability

All data generated or analyzed during this study are available in supplementary tables.
