# Supplementary material for: Escape behaviors in prey and the evolution of pennaceous plumage in dinosaurs
Source: Sci Rep. 2024 Jan 25;14:549. doi: 10.1038/s41598-023-50225-x (PMC10811223; doi:10.1038/s41598-023-50225-x)
Supplement: Supplementary file 1 — Supplementary Information. [file 41598_2023_50225_MOESM1_ESM.pdf]

Supplementary information

Escape behaviors in prey and the evolution of pennaceous plumage in dinosaurs

Authors: Jinseok Park[1], Minyoung Son[2,3], Jeongyeol Park[4], Sang Yun Bang[1], Jungmoon Ha[1], Hyungpil Moon[4*], Yuong-Nam Lee[2*], Sang-im Lee[5*], Piotr, G. Jablonski[1,6*]

[1] School of Biological Sciences, Seoul National University; Seoul, South Korea

[2] School of Earth and Environmental Sciences, Seoul National University; Seoul, South Korea

[3] Department of Earth and Environmental Sciences, University of Minnesota; Minneapolis, Minnesota, U.S.A.

[4] Department of Mechanical Engineering, Sungkyunkwan University; Suwon, South Korea

[5] Department of New Biology, DGIST; Daegu, South Korea

[6] Museum and Institute of Zoology, Polish Academy of Sciences, Warsaw, Poland

Supplementary text

Text S1

List of families marked in Figure S1 as “confirmed” (pink dots) and “suspected” (blue dots) visual flush-pursuers
Here we briefly describe the type of evidence available regarding flush-pursue foraging in these species and provide links to movie clips showing examples of their displays.

Visual flush-display foraging, which involves visual stimuli to make prey move/escape, is the most common, and we focus here only on this type. However, it has to be noted that some bird species in several families use non-visual flush strategies that work through tactile stimuli to make prey visible and available for attacks. Examples include the Scaly Thrush (*Zoothera dauma*[1]) and the Herring gull (*Larus argentatus*[2] cited in [3]).

It must be noted that the same or similar visual displays used in flush-display foraging may also serve as elements of antipredator reactions (e.g., as signals of vigilance) or in social interactions. To minimize the possibility of misclassifying these signals as flush-pursue displays, we critically evaluated the literature and **focused only on displays occurring exclusively during solitary foraging activities without the presence of other individuals (conspecifics or heterospecifics)**, as confirmed by video and audio in the clips. Additionally, we **excluded** situations in which birds might may felt **threatened** by predators, observers, or birdwatchers/videographers using predator calls or “pishing” sounds to attract the birds. Such situations can be recognized from the video and audio content by experienced field ornithologists who are attuned to various indicators for determining the context of the video, occasionally including other videos shot by the same person at the same location and time and also posted at the Macaulay library.

Also note that flush-pursuers use various foraging methods, including the flush-pursue strategy; no species rely exclusively on the flush-pursue strategy as their sole foraging strategy.

We list below all the families marked in Fig. S1. For some families, we do not have a clip with bird behavior, but we present relevant literature statements and results on which the classification is based. This list serves as an **overview of visual display behaviors during foraging**. A **complete phylogenetic analysis** of the evolutionary history of flush-displays will be addressed in a **separate paper**[4]. The numbers identifying the links to movie clips with examples of prey flush-displays refer to the numbers put on the schematic phylogeny in Fig. S1.

<u>Classification:</u>
Confirmed flush-pursuers (marked with * in the list below and with pink dots in Fig. S1) are species with solid evidence linking visual displays to pursuing prey during foraging. If a family includes **at least one confirmed flush-pursuer**, it is also marked with an asterisk (*; marked with pink dots in Fig. S1). In cases where a family contains multiple flush-pursuer species, only select examples are listed here. We include species for which the use of flush-pursue has been confirmed experimentally, as well as those for which only observational evidence exists.

Suspected flush pursuers (marked with ^ in the list below and with blue dots in Fig. S1) are actively foraging species (i.e., not sit-and-wait predators) with clear evidence for the use of displays during solitary foraging (excluding cases of possible communication among members of

a foraging group), but with weaker literature-based evidence for direct links between the display and pursuing prey, even though video evidence suggests the link may exist in the Macaulay library or on YouTube, and so on. All of these species perform **wing and/or tail displays** with various characteristics **in the absence of other individuals (conspecifics or heterospecifics) and in the context of foraging,** suggesting that the suspected function of flushing/disturbing prey. In some cases, other functions have also been suggested (e.g., signaling vigilance to predators), while the flush-pursue behavior was not clearly rejected (e.g., in Motacillidae).

The list below includes only one to several species per family, and a **comprehensive review of all avian species** that employ the **flush-pursue foraging** strategy as one among several foraging strategies will be a subject of a **separate review paper**. Nevertheless, this brief list below clearly illustrates that many bird families contain species that use the flush-pursue foraging. The species are grouped by family, and the families are ordered counterclockwise on the circular representation of the phylogenetic tree in Fig. S1.

Fifteen families are classified as containing “confirmed” flush pursuer(s), and another fifteen families are classified as containing “suspected” flush-pursuer(s). It is important to note that some of the suspected flush-pursuers may use behaviors like wing-flicking or other continuously repeated displays as signals of vigilance. This communication is less likely to be for interspecific communication because we attempted to include information about the behavior of solitary birds only. However, in some cases within the “suspected” category, there is clear evidence pointing to flush-pursue behavior. Even if we discard all information about “suspected” flush-pursuers, we still have 15 families with confirmed flush-pursuers. These families are distributed across different parts of the phylogenetic tree. The detailed reconstruction of ancestral states and evolutionary transitions between non-flush-pursue foraging styles and flush-pursue foraging will be addressed in our future research[4].

List of families with confirmed (*) and suspected (^) flush-pursuers:

*Pluvianidae

[*]**Egyptian plover (*Pluvianus aegyptius*)**

From the Birds of the World species account's[5] section “Diet and foraging”: *“catching flying insects on the run (these sometimes flushed by bird running with wings slightly spread).”*

^Turnicidae

[^]**Black-breasted Buttonquail (*Turnix melanogaster*)**

From the Birds of the World species account's[6] section “Diet and foraging”: *“Sometimes shades litter with outstretched wings while scratching.”* Scratching refers to activity performed during solitary foraging. It potentially may indicate that visual stimulus from outstretched wings affects the prey, although it may also serve to increase the visibility of the prey in the shadow of the wings.

*Cuculidae

[*]**Greater roadrunner (*Geococcyx californianus*)**

From the Birds of the World species account's[7] section “Diet and foraging”: *“Frequently flashes white spots visible on open wings to startle or flush prey.”*

Link 1) https://www.youtube.com/watch?v=qmXGDWkZSek, © Kat Avila, uploaded on 4th October 2017; in this clip, the species uses relatively simple wing opening and closing movements during foraging.

*__Striped Cuckoo (*Tapera naevia*)__ uses its alulae in flush-pursue foraging ([8] section "Diet and foraging").
Link 2) https://macaulaylibrary.org/asset/201222491; the species uses alulae spreading and folding movements during foraging in these clips.

^Eurypygidae
^**Sunbittern (*Eurypyga helias*)**
Link 3) https://macaulaylibrary.org/asset/ML201989021; this clip shows the species using one wing as if to affect the behavior of prey, maybe by affecting the direction of the prey escape/move, which is followed by an attack. However, we cannot exclude the possibility that this asymmetric display's function was to simply provide shadow to the area where the prey may be located.

***Trogonidae**
*__Malabar Trogon (*Harpactes fasciatus*)__
It has been described that this species uses a flush-pursue strategy to flush prey out from hiding places and then pursue the prey[9].

^Acanthisittidae
^**Rifleman (*Acanthisitta chloris*)**
From the Birds of the World species account's[10] section "Diet and foraging": *"Restless forager along trunks and branches; wings constantly flicked."*

^Pittidae
^**Ornate Pitta (*Pitta concinna*)**
Link 4) https://macaulaylibrary.org/asset/201662871; in this clip, the species spreads its wings during foraging and then apparently pounces on prey; it also uses wing-flicking As pittas are challenging to observe among the thick undergrowth of their natural habitats, no more evidence corroborating or rejecting the use of flush-pursue foraging in this taxon is available at the moment.

***Oxyruncidae**
*__Tawny-breasted Flycatcher (*Myiobius villosus*)__
From the Birds of the World species account's[11] section "Diet and foraging": *"droops wings, often pivots, uses flush-and-chase strategy."*

***Tyrannidae**
*__Golden-faced Tyrannulet (*Zimmerius chrysops*)__
This species has been mentioned using the flush-pursue strategy[12].
From the Birds of the World species account[13]: *"Actively hops, tail often cocked slightly above horizontal."*

***Thamnophilidae**

[*]Dot-winged Antwren (*Microrhopias quixensis*)
This species has been confirmed to use flush-pursue foraging[14].
Link 5) https://macaulaylibrary.org/asset/200781261; in this clip, the species flicks its wings during foraging through the foliage.
[*]Rufous-backed Stipplethroat (*Epinecrophylla haematonota*)
This species has been confirmed using flush-pursue foraging[14].

***Furnariidae**
[*]Plain-brown Woodcreeper (*Dendrocichla fuliginosa*)
The flush-pursuing with wing-flashing displays behavior has been noted by observers[15]: "*Plain-brown Woodcreepers frequently use wing-flashing when prey stops and is concealed. The bird moves to the trunk where prey disappeared and briefly flashes one wing widely along the surface of the trunk. On slender trunks the bird may simultaneously sidle and peer around the trunk from the opposite direction, so that it will run into prey fleeing the wing.*"
[*]White-chinned Woodcreeper (*Dendrocincla merula*)
The flush-pursuing with wing-flashing displays behavior has been observed[15]: *"I recorded successful wing-flashing to flush prey by a White-chinned Wood-creeper at Cashibococha, Peru."*
[*]Tawny-winged Woodcreeper (*Dendrocincla anabatina*)
As in the White-chinned woodcreeper, the flush-pursuing with wing-flashing displays behavior has been noted[15]: *"Tawny-winged Woodcreepers flash the wings even more frequently than do Plain-brown Woodcreepers. Perhaps the conspicuous tawny wing patches of the Tawnywing and the yellow undersides of the wings of all three species are adaptations for flushing prey."*

***Rhipiduridae**
[*]White-browed Fantail (*Rhipidura aureola*)
From the Birds of the World species account's[16] section "Diet and foraging": *"Flushes prey by restlessly twisting and turning along branches and tree trunks, flicking wings open and fanning tail."*
Link 6) https://www.youtube.com/watch?v=ifxkGriCaXM © Shirishkumar Patil, uploaded on 22nd April 2014; the species spreads its tail and flicks wings in this clip.
[*]Willie-wagtail (*Rhipidura leucophrys*)
From the Birds of the World species account's[17] section "Diet and foraging": *"Forages mostly by flycatching, flush-pursuit and gleaning."*
Link 7) https://www.youtube.com/watch?v=6B0YdC1FxBE © oceanbirds1, uploaded on 24th July 2015; the species flush prey by fanning/wagging tail (sidewise), and flicking wings in this clip.
[^]White-throated Fantail (*Rhipidura albicollis*)
Link 8) https://macaulaylibrary.org/asset/201032211; the species shows tail fanning and wing dropping during foraging in this clip.

***Monarchidae**
[*]Blue-headed Crested-Flycatcher (*Trochocercus nitens*)
From the Birds of the World species account's[18] section "Diet and foraging": *"Moves actively, with tail held fully fanned and wings spread and drooped. Rapidly beats wings, or stretches*

wings wide and twists tail, to disturb prey; snaps up insects in air in short sally or circular descending flight."

[*]African Paradise-flycatcher (*Terpsiphone viridis*)

The flush-pursuing with fan-fail displays behavior has been observed ([19] section "Diet and foraging").

***Stenostiridae**

[*]Yellow-bellied Fairy-fantail (*Chelidorhynx hypoxanthus*)

From the Birds of the World species account's[20] section "Diet and foraging": *"Prey flushed by fluttering, and captured in aerobatic sallies."*

[^]African Blue Flycatcher (*Elminia longicauda*)

From the Birds of the World species account's[21] section "Diet and foraging": *"Actively forages in canopy, with wings held half-drooped and tail continually spread."*, in the manner similar to *Myioborus* redstarts in which this behavior has been experimentally proved to be used in flush-pursue foraging.

Link 9) https://macaulaylibrary.org/asset/201957981; in this clip, the species droops wings and spreads tail.

^Paridae

[^]Yellow-browed Tit (*Sylviparus modestus*)

From the Birds of the World species account in the section's[22] "Diet and foraging": *"Restless, flits about through foliage; agile movements and behavior, including nervous wing-flicking, reminiscent of those of a leaf-warbler."* Wing-flicking in leaf-warblers (Phylloscopidae) has been suggested to be linked with flush-pursue foraging.

***Scotocercidae**

[*]Chestnut-capped Flycatcher (*Erythrocercus mccallii*)

From the Birds of the World species account's[23] section "Diet and foraging": *"dislodges insects by flicking wings and making wide sweeps with tail spread; also makes short dashing flights in pursuit of insects."*

Link 10) https://macaulaylibrary.org/asset/201274401; the species flicks wings, spreads tail, and pivots its body in this clip.

^Phylloscopidae

Some species in this family flick wings during foraging nearly constantly, and it has been suggested[24], however not fully proven, that this may flush prey that is subsequently pursued. While flush-pursuing is possible and has not been entirely rejected, the behavior and bright plumage colors in Phylloscopidae may be under strong selection for communication[25]. Examples of wing-flicking:

[^]Common Chiffchaff (*Phylloscopus collybita*)

From the Birds of the World species account's[26] section "Diet and foraging": *"Frequently dips tail when foraging."*

Link 11) https://macaulaylibrary.org/asset/201574941; the species flicks its wings fast and moves its tail up and down vertically in this clip of a foraging individual.

[^]Willow Warbler (*Phylloscopus trochilus*).

Link 12) https://macaulaylibrary.org/asset/201189341; the species flicks its wings fast on the ground in this clip.

^Pycnonotidae

^Fischer's Greenbul (*Phyllastrephus fischeri*)

From the Birds of the World species account's[27] section "Diet and foraging": *"Flicks tail and wings"* during foraging.

^Northern Brownbul (*Phyllastrephus strepitans*)

From the Birds of the World species account's[28] section "Diet and foraging": *"Flicks wings and tail constantly, both when foraging and when perched."*

^Acrocephalidae

^Upcher's Warbler (*Hippolais languida*)

From the Birds of the World species account's[29] section "Diet and foraging": *"Waving tail to the sides, flicks wings rapidly while foraging, and sometimes stretches one wing straight out."*

^Cisticolidae

^Chestnut-throated Apalis (*Apalis porphyrolaema*)

From the Birds of the World species account's[30] section "Diet and foraging": *"While foraging, often sways body from side to side, with wings held drooped, and tail erect and fanned, and flicked sideways, in manner of a monarch-flycatcher (Monarchidae), which family contains confirmed flush-pursuers."*

^Black-collared Apalis (*Oreolais pulcher*)

From the Birds of the World species account's[31] section "Diet and foraging": *"Has habit of extravagantly wagging its raised tail from side to side"* during foraging.

^Cricket Longtail (*Spiloptila clamans*)

From the Birds of the World species account's[32] section "Diet and foraging": *"Tail continuously rotated and flirted"* during foraging.

^Common Tailorbird (*Orthotomus sutorius*)

From the Birds of the World species account's[33] section "Diet and foraging": *"Hopping with tail held cocked and flicked from side to side"* during foraging.

Link 13) https://macaulaylibrary.org/asset/201469261; a foraging individual flicks its wings and hops with cocked tail in this clip.

^Pnoepygidae

^Scaly-breasted Cupwing (*Pnoepyga albiventer*)

From the Birds of the World species account's[34] section "Diet and foraging": *"Frequently flicks its wings while foraging."*

Link 14) https://macaulaylibrary.org/asset/339321481; a solitary foraging individual actively flicks its wings in this clip.

^Timaliidae

^Golden Babbler (*Cyanoderma chrysaeum*)

Link 15) https://macaulaylibrary.org/asset/201405711

Link 16) https://macaulaylibrary.org/asset/201976161; the species actively moves and flicks its wings in these clips of solitary foraging birds.

***Sittidae**

***Velvet-fronted Nuthatch (*Sitta frontalis*)**

From the Birds of the World species account's[35] section "Diet and foraging": *"Vigorous wing-flapping observed, apparently an attempt to flush insects from face of tree trunks."*

Link 17) https://macaulaylibrary.org/asset/426344141, the species spreads its wings and rapidly folds it in this clip.

^Polioptilidae

^Blue-grey Gnatcatcher (*Polioptila caerulea*)

From the Birds of the World species account's[36] section "Diet and foraging": *"Moves tail constantly, which may flush unseen prey."*

Link 18) https://macaulaylibrary.org/asset/330549521; the species swings and rapidly moves its tail in the vertical axis in this clip.

^Tichodromidae

^Wallcreeper (*Tichodroma muraria*)

Link 19) https://macaulaylibrary.org/asset/723326; the species spreads and folds its wings fast on the rock during foraging for prey that seems to be pecked from the substrate suggesting a possible role of flicking in revealing prey's presence to the bird, however, the suggested function of flicking in professional literature, based on observations only, is "signaling" in this clip.

***Mimidae**

***Northern mockingbird (*Mimus polyglottos*)**

Its flush-display consists of stereotypically performed hitches and pauses[37].

Link 20) https://www.youtube.com/watch?v=boZz0ECyYEQ © Linzy's Vids, uploaded on 17th October 2016.

^Muscicapidae

^Rufous-tailed scrub robin (*Cercotrichas galactotes*)

Link 21) https://macaulaylibrary.org/asset/200888551; the species uses its wings and tail movements simultaneously during foraging in these clips.

^Black Scrub-Robin (*Cercotrichas podobe*)

Link 22) https://macaulaylibrary.org/asset/215171291; the species moves on the ground while spreading its tail and wings. It uses wings and tail separately and sometimes uses both in this clip.

^Motacillidae

^Gray Wagtail (*Motacilla cinerea*)

Link 23) https://macaulaylibrary.org/asset/201514801; the species wags its tail while foraging in this clip. Flushing prey was not rejected as a possible function of wagging, but it may be a signal of vigilance against predators[38].

***Thraupidae**

***Guira Tanager (*Hemithraupis guira*)**

This species uses the flush-pursue strategy[12]. But there is no detailed description of the display characteristics.

***Parulidae**

[*]**Slate-throated Redstart (*Myioborus miniatus*)** hops and pivots its body through foliage white spreading its tail and wings[39].

Link 24) https://macaulaylibrary.org/asset/201955691; the species spreads its wings and tail, combined with body pivoting in this clip.

[*]**Painted Redstart (*Myioborus pictus*)** uses tail and wing spreading and also body movements (e.g., pivoting) during foraging[40–42].

Link 25) https://macaulaylibrary.org/asset/464937; the species spreads its wings and tail, combined with body pivoting and hopping in this clip.

[*]**Collared Redstart (*Myioborus torquatus*)**

From the Birds of the World species account[43]: *"sometimes advancing through foliage or along branches with the wings drooped and the tail fanned, exposing the white outer rectrices; it pursues small insects that are flushed by its approach."*

Link 26) https://macaulaylibrary.org/asset/201828251; the species spreads its wings and tail in this clip.

Supplementary figures

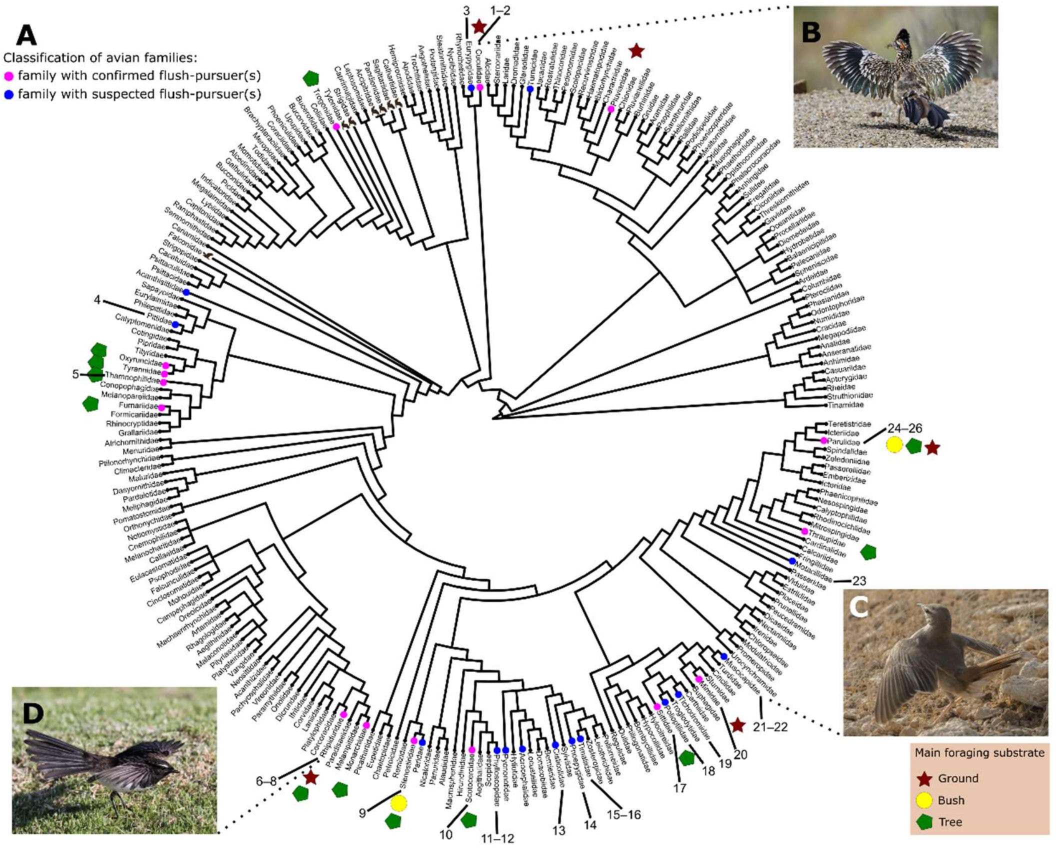

Figure S1. Diversity of extant avian flush-pursuers. (**A**) Distribution of flush-pursuers among 248 avian families across diverse avian taxa. Pink dots indicate families containing at least one species with evidence of using flush-pursue foraging and classified as “confirmed” flush-pursuers (Text S1). Blue dots indicate additional families containing at least one “suspected” flush-pursuer (Text S1) defined as actively foraging species (i.e., not sit-and-wait predator) with evidence of using displays during foraging movements but with weaker evidence for direct links between the display and pursuing of flushed prey (albeit video evidence suggesting this link may exist). The symbols of the brown star, yellow circle, and green pentagon denote the main foraging substrate of confirmed flush-pursuers within a family, with each symbol indicating species that primarily forage on the ground, in bushes, and in trees, respectively. The bird-symbol at the tip of the phylogeny indicates families of predatory birds, including hawks, eagles, vultures, owls, and falcons. This consensus tree was built using a tree set obtained from BirdTree.org. (**B–D**) Photos illustrating examples of displays of ground-foraging flush-pursuers: Greater Roadrunner (*Geococcyx californianus*), Rufous-tailed Scrub-Robin (*Cercotrichas galactotes*), Willie-wagtail (*Rhipidura leucophrys*). We used the following recordings from the Macaulay Library at the Cornell Lab of Ornithology: ML98307051, ML366333971, ML205494131. This figure concerns Fig. 1G. The numbers next to each family correspond to the numbers given to the video links listed in Text S1. A comprehensive review of flush-pursuing birds will be the subject of a separate review paper[4].

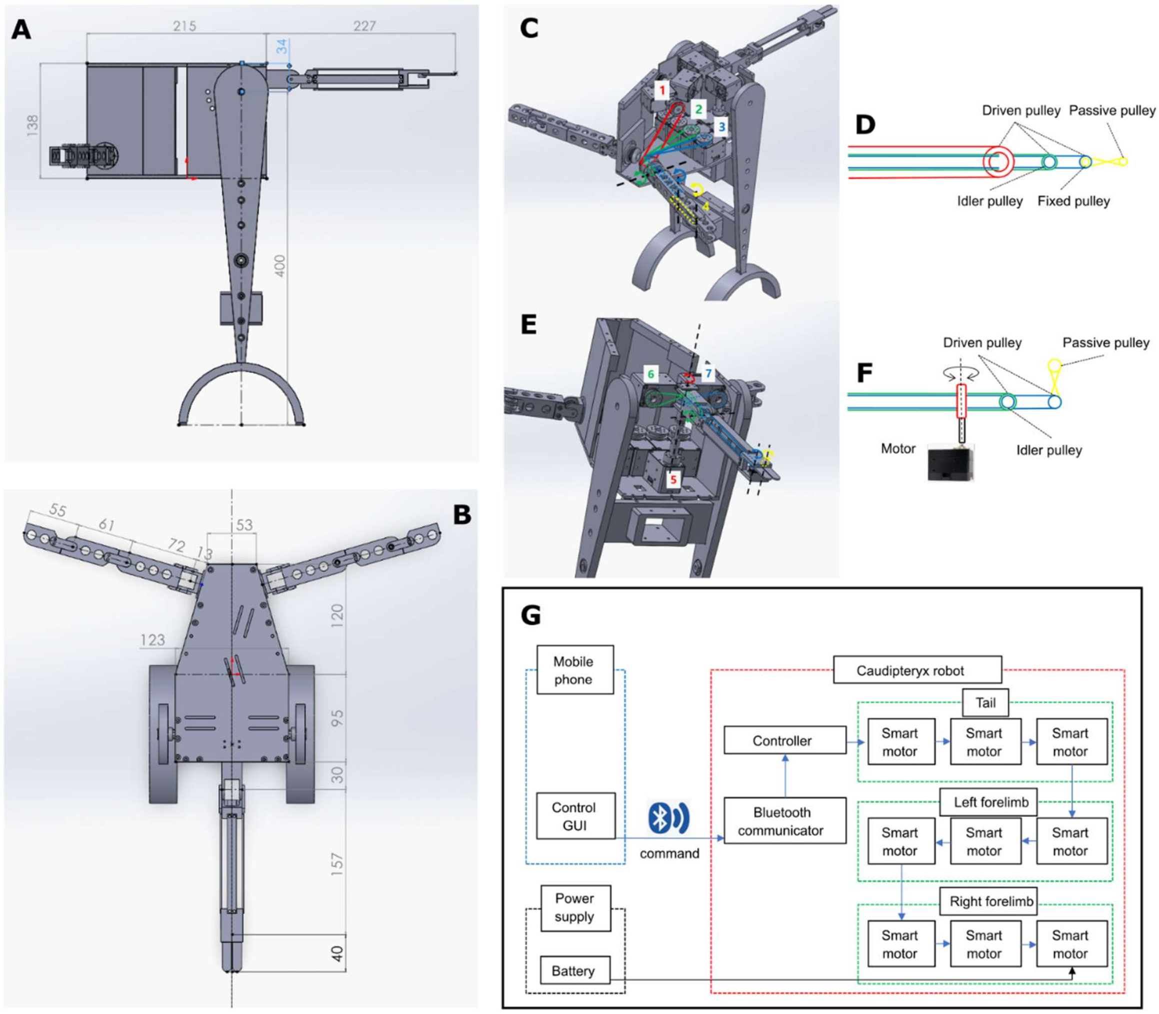

Figure S2. Hardware and control system of the *Caudipteryx* robot (Robopteryx). (**A**) Side view of the CAD model used for building Robopteryx. (**B**) Top view of the CAD model used for building Robopteryx. The unit length is in mm. (**C** and **D**) For forelimb motion control, two fishing lines (thickness: 0.47 mm, tensile strength: 45 kg) and a belt connected to a motor (XM430-W210-R, Robotis) are used as tendons to control pitch (green), yaw (blue), and roll (red) rotations. The fishing line (blue) connected with motor no.3 also implements rotating at the elbow joint. An additional fishing line (yellow) is used for passive rotation of the wrist. (**E** and **F**) Tail motion is controlled using a motor (no.6) with a fishing line (green) for pitch motion. We also implement yaw rotation (motor no.5) and spreading of the tail tip (blue) in the robot. (**G**) The schematic diagram of the robot control system. The controller (OpenCM9.04-C, Robotis) receives operation commands created from a mobile phone through the Bluetooth communicator (BT-410, Robotis). The controller is connected to a series of smart motors. An external battery (LIPO Battery LB-020, Robotis) supplies power.

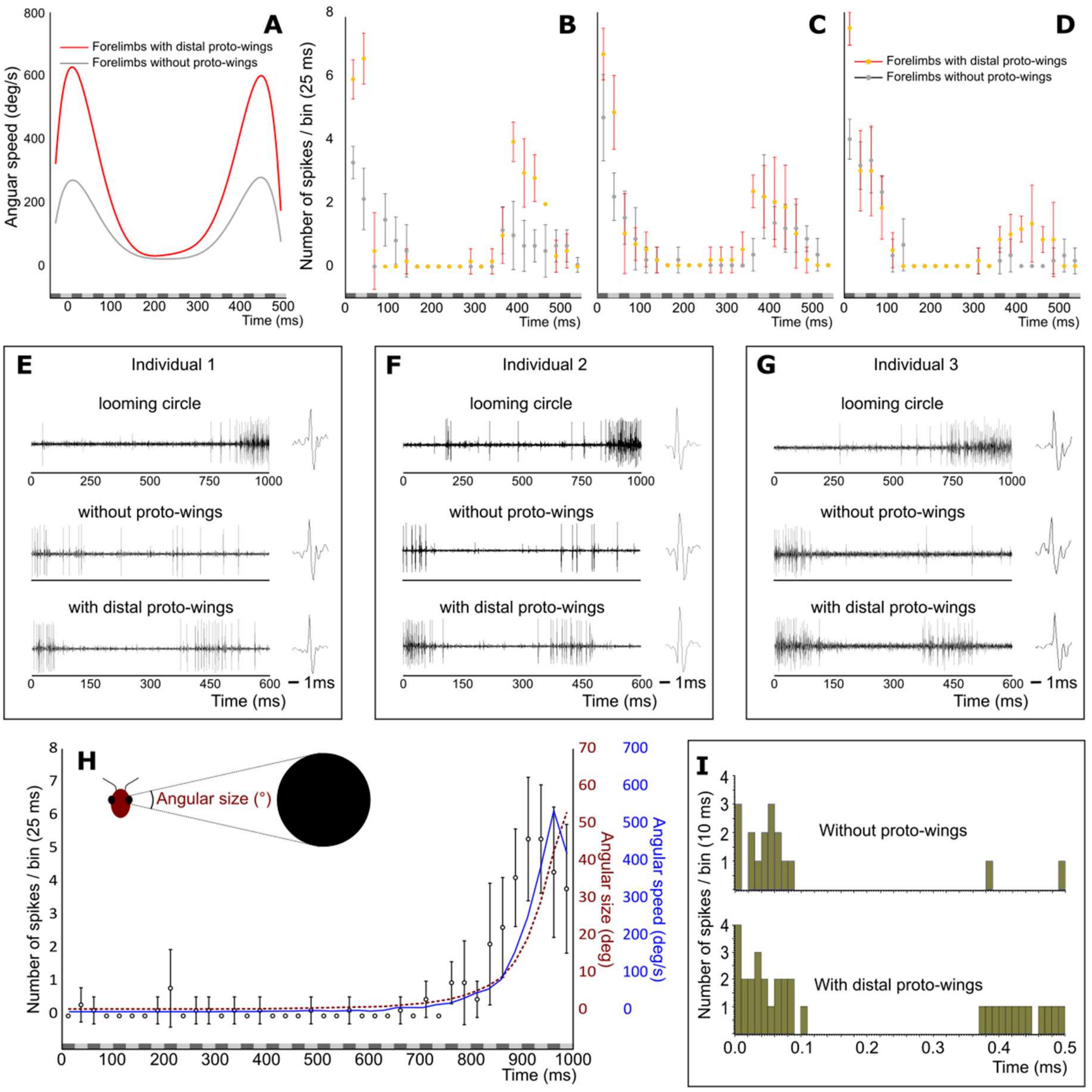

Figure S3. Additional results of neurophysiological experiments. (**A**) Angular speed calculated from the forelimb tips' movements in the *Caudipteryx* animations (Video S1, parts 4 and 5). (**B**–**D**) The firing rate (no. of spikes/25 ms bin; average ± SD) of the looming-sensitive escape pathway from each of three individuals in response to the animations of forelimb display without (gray bar; n = 6) and with (red bar; n = 6) distal proto-wings. All records from three individuals are used in Fig. 1G. Bins are marked as gray bars along the horizontal axis. (**E**–**G**) Examples of recordings from the grasshopper' LGMD/DCMD looming-detective pathway from each of three individuals in response to animations: a looming circle; forelimb movement without proto-wings; forelimb movement with proto-wings. A spike next to a recording is an example from that recording. Spike shapes slightly differed between individual grasshoppers (especially in terms of the amplitude of the lower and upper part of the spike), but the spike shapes were similar between the looming circle and the animations of flush displays within the same individual grasshopper. A comprehensive study of the neurophysiological responses to a full variety of hypothetical displays by flush-pursuing dinosaurs will be the subject of a separate

paper. The spike data are shown in Tables S5 and S6. (**H**) The firing rate in response to a looming circle. The firing rate (no. of spikes/25 ms bin; average ± SD) of the grasshopper looming-sensitive escape pathway in response to a looming circle animation [n = 6 (2 recordings from each of three individuals)]. The approaching speed was 6 m/s. The right-side Y-axis shows the stimulus angular size (deg; dotted burgundy line) and speed (deg/s; solid blue line) of the looming circle. Bins are marked as gray bars along the horizontal axis. The spike data are shown in Table S9. (**I**) Examples of the firing rate of the escape pathway analyzed with a bin size of 10 ms to show that even with a short bin size, the peak value of spiking frequency occurs right at the outset of the flush display animation. The remaining analyses were conducted using a bin size of 25 ms to decrease random variation among bins.

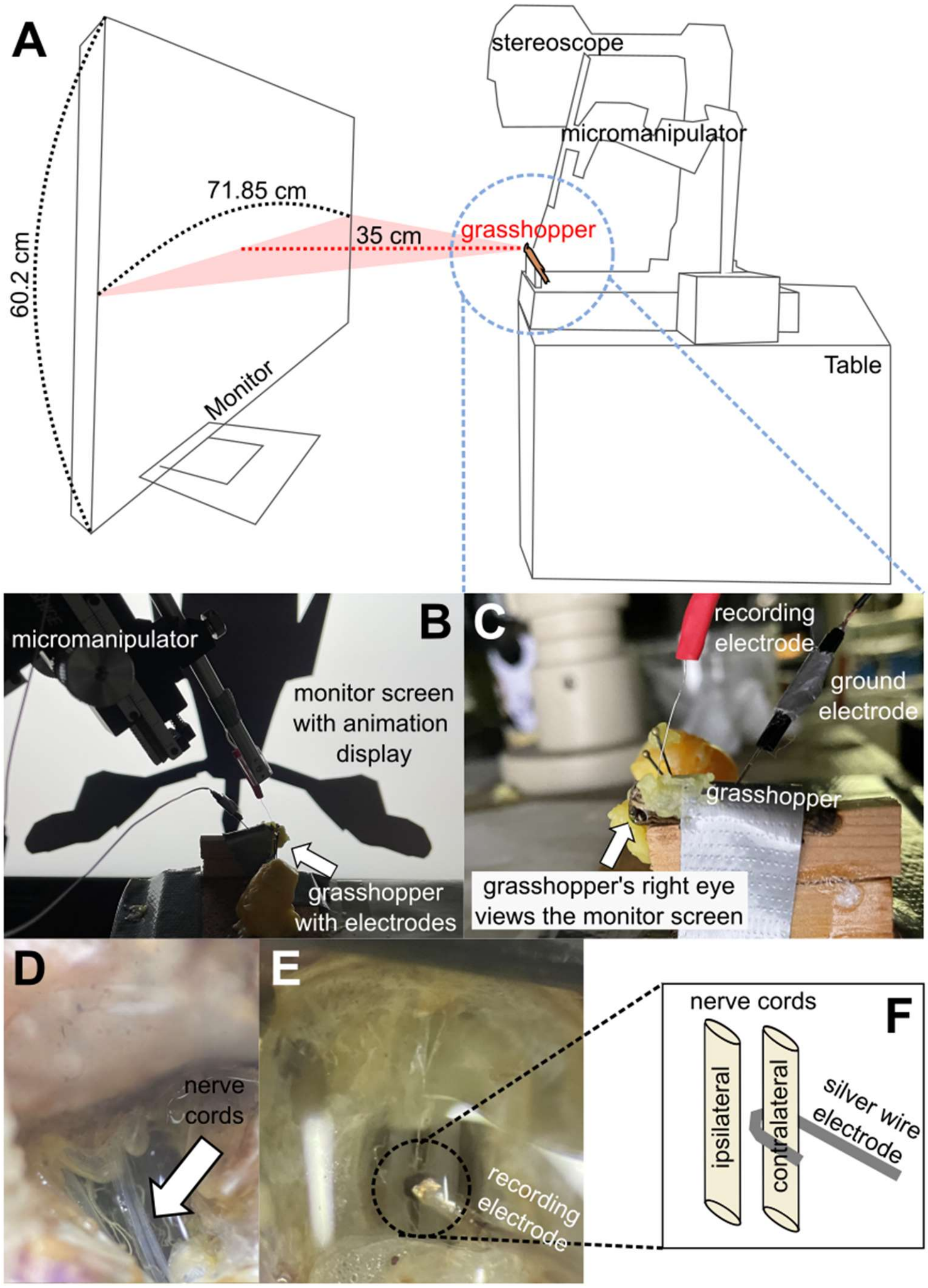

Figure S4. Experimental setup for neurophysiological experiments. (**A**) A schematic view of the experimental setup for neurophysiological experiments. A grasshopper and equipment are placed on a table. The distance between the monitor and the grasshopper's right eye is set to 35 cm. (**B**) *Caudipteryx* animation is displayed on the monitor. (**C**) The silver wire hook-electrode (red, serving as the recording electrode) is wrapped around the contralateral ventral nerve cord, and a pin (black, acting as the ground electrode) is inserted into the grasshopper's abdomen, connecting to the ground. (**D**) An arrow indicates the ventral nerve cords of the grasshopper. (**E** and **F**) A dotted circle indicates the point of the ventral nerve cord where the electrode is hooked to the contralateral nerve cord.

Supplementary tables

Table S1. Results of experiment 1. The effect of the robot's motor sound (Treatment 1-1) and the presence of proto-wings (Treatment 1-3 *vs.* 1-2) on escape distance and frequency by grasshoppers during a step-wise approach of the robot starting from 100 cm with maximally up to five stops with flush displays at 100, 80, 60, 40, and 20 cm to the grasshopper. The approach ended either at 20 cm or when the grasshopper escaped (details in Methods Part 4 Experiment 1; results in Fig. 2B). *P*-values for each pair of experimental conditions were (1-1) vs. (1-2) < 0.001, (1-1) vs. (1-3) < 0.0001, (1-2) vs. (1-3) < 0.0001 (Dunn's test with Bonferroni correction). The number in parentheses indicates the number of grasshoppers tested with the displaying robot placed directly in front or behind them. Additional analysis of the data set, excluding these situations, showed similar results [Dunn's test with Bonferroni correction, *P* for (1-1) vs. (1-2) < 0.001, (1-1) vs. (1-3) < 0.0001, (1-2) vs. (1-3) < 0.0001]. This table concerns Fig. 2B.

Test distance	Experimental condition		
	Motor sound playback without movements Treatment (1-1)	Flushing movements without the proto-wings Treatment (1-2)	Flushing movement with the proto-wing Treatment (1-3)
100 cm	0	0	1
80 cm	0	1 (1)	0
60 cm	0	0	9 (2)
40 cm	0	9 (2)	15 (2)
20 cm	2 (1)	10	17 (8)
No response	44 (6)	23 (7)	3 (1)
Total	46 (7)	43 (10)	45 (13)

Table S2. Results of experiment 2. The number of grasshoppers that escaped at each test distance and the number of grasshoppers that did not respond to the experimental conditions, even at a distance of 35 cm. We presented the robot to the grasshoppers in three experimental conditions: (2-1) without proto-wings; (2-2) with proximally located proto-wings presented; (2-3) with distally located proto-wings. None of the grasshoppers responded to the robot's flushing movements at a distance of 70 cm. Therefore, we only used responses at 35 cm for statistical comparisons among the treatments. *P*-values for each pair of experimental conditions are (2-1) vs. (2-2) = 0.02, (2-1) vs. (2-3) < 0.0001, (2-2) vs. (2-3) < 0.0001 [Chi-square test with Bonferroni correction]. The number in parentheses indicates the number of grasshoppers tested with the displaying robot placed directly in front or behind them. Additional analysis of the data set, excluding these data points, showed similar results [Chi-square test with Bonferroni correction, *P* for (2-1) vs. (2-2) = 0.02, (2-1) vs. (2-3) < 0.0001, (2-2) vs. (2-3) < 0.001]. This table concerns Fig. 2C.

Test distance	Experimental treatments		
	No proto-wings Treatment (2-1)	Proximal proto-wings Treatment (2-2)	Distal proto-wings Treatment (2-3)
70 cm	0	0	0
35 cm	1	10	27 (1)
No response	29 (2)	20 (1)	3
Total	30 (2)	30 (1)	30 (1)

Table S3. Results of experiment 3. The number of grasshoppers that escaped at each test distance and the number of grasshoppers that did not respond in experimental conditions, even at a distance of 40 cm. We presented the robot to the grasshoppers in two experimental treatments: (3-1) plain black distal proto-wings; (3-2) white-patched distal proto-wings. None of the grasshoppers responded to the robot's flushing movements at a distance of 60 cm. Therefore, we used only responses at 40 cm for statistical comparisons among the treatments. *P*-value for (3-1) vs. (3-2) < 0.01 (Chi-square test with Yates' continuity correction). The number in parentheses indicates the number of grasshoppers tested with the displaying robot placed directly in front or behind them. Additional analysis of the data set, excluding these points, showed similar results. (Chi-square test with Yates' continuity correction, $P < 0.001$). This table concerns Fig. 2D.

	Experimental treatments	
Test distance	Plain black distal proto-wings Treatment (3-1)	White-patched distal proto-wings Treatment (3-2)
60 cm	0	0
40 cm	3 (1)	15 (1)
No response	27	15 (4)
Total	30 (1)	30 (5)

Table S4. Results of experiment 4. The number of grasshoppers that escaped at each test distance and the number of grasshoppers that did not respond to experimental treatments, even at a distance of 60 cm in experiment 4. We presented the robot to the grasshoppers in three experimental tratments: (4-1) without caudal plumage; (4-2) with caudal plumage of the size imitating *Caudipteryx* fossil; (4-3) with caudal plumage twice the area of (4-1). None of the grasshoppers responded to the robot's flushing movements at a distance of 80 cm. Therefore, we only used responses at 60 cm for statistical comparisons among the treatments. *P*-values for each pair of experimental conditions were (1-1) vs. (1-2) = 0.02, (1-1) vs. (1-3) < 0.0001, (1-2) vs. (1-3) < 0.01 (Dunn's test with Bonferroni correction). The number in parentheses indicates the number of grasshoppers tested with the displaying robot placed directly in front or behind them. Additional analysis of the data set, excluding these points, showed similar results [Dunn's test with Bonferroni correction, *P* for (4-1) vs. (4-2) = 0.03, (4-1) vs. (4-3) < 0.0001, (4-2) vs. (4-3) < 0.01)]. This table concerns Fig. 2E.

Test distance	Experimental treatments		
	Without caudal plumage Treatment (4-1)	Normal-sized caudal plumage Treatment (4-2)	Twice-sized caudal plumage Treatment (4-3)
80 cm	0	3	11
60 cm	0	10	16
No response	30 (2)	35 (1)	21
Total	30 (2)	48 (1)	48

Table S5. Data set collected in neurophysiological experiments: treatment "without proto-wings". The number of recorded DCMD spikes in response to the "without proto-wings" animation for three individuals. The spike numbers are summed up in every bin (25 ms). We recorded the neural response six times for each individual. This table concerns Figs. 2G and S3B–D.

	Individual 1						Individual 2						Individual 3					
Bin order	Record number in order of presentation						Record number in order of presentation						Record number in order of presentation					
(25 ms)	R1	R2	R3	R4	R5	R6	R1	R2	R3	R4	R5	R6	R1	R2	R3	R4	R5	R6
1	4	3	4	3	3	3	6	5	6	5	3	3	4	5	4	4	4	3
2	2	4	1	2	2	2	1	2	2	2	3	3	4	3	4	2	3	3
3	0	0	0	0	0	0	3	2	1	1	1	1	5	3	4	3	3	2
4	2	2	2	0	1	2	1	2	0	0	2	0	2	3	2	2	3	2
5	2	0	0	1	1	1	0	0	1	0	0	0	0	0	1	1	0	0
6	0	0	0	1	2	0	0	1	0	0	0	0	0	2	0	1	1	0
7	0	0	0	0	0	0	0	0	0	0	0	0	0	0	0	0	0	0
8	0	0	0	0	0	0	0	0	0	1	0	0	0	0	0	0	0	0
9	0	0	0	0	0	0	0	0	0	0	0	0	0	0	0	0	0	0
10	0	0	0	0	0	0	0	0	0	0	0	0	0	0	0	0	0	0
11	0	0	0	0	0	0	0	0	0	0	0	0	0	0	0	0	0	0
12	0	0	0	0	0	0	0	0	0	0	0	0	0	0	0	0	0	0
13	0	0	0	0	0	0	0	0	0	0	0	0	0	0	1	0	0	0
14	0	0	0	0	0	0	0	0	0	0	0	0	0	0	0	0	0	0
15	2	1	2	1	1	0	1	0	0	1	0	0	0	1	0	0	0	0
16	1	1	1	0	3	0	3	3	3	3	0	1	1	0	0	0	1	0
17	1	2	1	0	0	0	1	2	1	1	2	1	0	0	0	0	0	0
18	0	1	0	1	1	1	1	0	2	1	2	1	0	0	0	0	0	0
19	0	0	1	0	2	0	1	2	1	0	1	2	0	0	0	0	0	0
20	1	1	1	0	0	1	1	1	1	1	1	0	1	0	0	0	0	0
21	1	1	1	1	0	0	0	1	1	0	0	0	0	1	0	0	0	1
22	0	0	0	0	0	0	0	0	0	0	0	0	0	0	1	0	0	0

Table S6. Data set collected in neurophysiological experiments: treatment "with proto-wings". The number of recorded DCMD spikes in response to the "with distal proto-wing" animation for three individuals. The spike numbers are summed up in every bin (25 ms). We recorded the neural response six times for each individual. This table concerns Figs. 2G and S3B–D.

	Individual 1						Individual 2						Individual 3					
Bin order	Record number in order of presentation						Record number in order of presentation						Record number in order of presentation					
(25 ms)	R1	R2	R3	R4	R5	R6	R1	R2	R3	R4	R5	R6	R1	R2	R3	R4	R5	R6
1	7	6	5	6	6	6	8	7	6	6	7	6	8	8	8	7	7	7
2	6	7	8	6	6	7	6	5	5	6	3	4	5	4	2	3	2	2
3	3	0	0	0	0	0	3	1	2	0	0	0	4	5	3	3	2	1
4	0	0	0	0	0	0	1	1	1	1	0	0	3	2	3	1	1	1
5	0	0	0	0	0	0	0	1	1	0	1	0	1	0	1	1	0	0
6	0	1	0	0	0	0	0	0	1	0	0	0	0	0	0	0	0	0
7	0	0	0	0	0	0	0	0	0	0	0	0	0	0	0	0	0	0
8	0	0	0	0	0	0	0	0	0	0	0	0	0	0	0	0	0	0
9	0	0	0	0	0	0	0	0	0	0	0	0	0	0	0	0	0	0
10	0	0	0	0	0	0	0	0	0	0	0	0	0	0	0	0	0	0
11	0	0	0	0	0	0	0	0	0	0	0	1	0	0	0	0	0	0
12	0	1	0	0	0	0	0	1	0	0	0	0	0	0	0	0	0	0
13	0	0	0	0	0	0	0	0	0	0	0	1	0	0	0	0	0	1
14	0	0	0	0	1	0	1	0	0	0	1	1	0	0	0	0	0	0
15	1	0	1	2	2	0	2	2	2	3	3	2	1	1	1	0	1	1
16	4	3	4	4	4	5	2	2	4	1	2	2	2	1	0	1	1	1
17	3	3	3	2	5	2	4	3	1	2	0	2	2	1	1	1	1	1
18	2	3	3	3	2	4	3	1	3	3	1	0	3	2	0	1	2	0
19	2	2	2	2	2	2	3	1	1	0	1	0	1	1	1	1	1	0
20	0	0	0	1	0	1	0	1	0	0	0	0	3	0	1	0	1	0
21	1	1	0	0	1	0	0	0	0	0	0	0	0	0	0	0	0	0
22	0	0	0	0	0	0	0	0	0	0	0	0	0	0	0	0	0	0

Table S7. Linear model for explaining variations in the size of two peaks in DCMD firing rate. The model includes two fixed effects: treatment (proto-wings presence vs. absence) and individual ID (with three levels). The values in the table represent the effect estimate for each variable, standard error, t-value, and p-value.

Peak type	Coefficients	Estimate	Standard Error	t-value	p-value
Frist peak	Intercept	6.6111	0.2940	22.489	< 2E-16
	Treatment, proto-wings absence	-2.8899	0.2940	-9.827	3.47E-11
	Individual ID, number 2	0.6667	0.3600	1.852	0.0733
	Individual ID, number 3	0.5000	0.3600	1.389	0.1745
Second peak	Intercept	3.6389	0.2715	13.405	1.12E-14
	Treatment, proto-wings absence	-1.2778	0.2715	-4.707	4.65E-05
	Individual ID, number 2	-0.1667	0.3325	-0.501	0.62
	Individual ID, number 3	-1.7500	0.3325	-5.264	9.23E-06

Table S8. Estimated angular ranges (min value – max value) of possible movements of *Caudipteryx*'s forelimb. The ranges were inferred from the nearest species [more primitive (*Acrocanthosaurus*) and advanced species (*Bambiraptor*)]. Details are described in Methods part 3. This table concerns Fig. 3G.

Angle	Theropod species		
	Primitive species: *Ancrocanthosaurus*[44]	Advanced species: *Bambiraptor*[45]	**Hypothetical ranges in *Caudipteryx* [range of possible minimal value] ~ [range of possible maximal value]**
Shoulder (S)	-19° – 144°	2° – 123°	**-19°~2° – 114°~123°**<br>= *Acrocanthosaurus~Bambiraptor – Acrocanthosaurus~Bambiraptor*
Elbow (E)	104° – 159°	55~59° – 127~136°	**55° – 136°**<br>= *Bambiraptor*<br>Maybe it could fold up to 30° [46]
Wrist (W)	?	104° – 167°	**0° – ~180°**<br>= can fold like current birds –<br>cannot be fully unfolded due to its joint structure, < 180°
Lifting (L)	?	~88°	**? – 88°**<br>= maximum value in *Bambiraptor*<br>*Caudipterix* group could not raise the arm horizontally due to its joint structure

Table S9. Response of the LGMD/DCMD pathway to an animation of a simple looming stimulus. The number of recorded DCMD spikes in response to the "looming circle" animation for three individuals. The spike numbers are summed up in every bin (25 ms). We recorded the neural response two times for each individual, both at the beginning and at the end of the recording session. This table concerns Fig S3H.

	Individual 1		Individual 2		Individual 3	
Bin order	Record number		Record number		Record number	
(25 ms)	R1	R2	R1	R2	R1	R2
1	0	0	0	0	0	0
2	1	1	0	0	0	0
3	0	0	0	0	1	0
4	0	0	0	0	0	0
5	0	0	0	0	0	0
6	0	0	0	0	0	0
7	0	0	0	0	0	0
8	0	0	1	0	0	0
9	0	0	3	0	1	1
10	0	0	0	0	0	0
11	0	0	0	0	0	1
12	0	0	0	0	0	1
13	0	0	0	0	0	0
14	0	0	0	0	0	1
15	0	0	0	0	0	0
16	0	0	1	0	0	0
17	0	0	0	0	0	0
18	0	0	0	0	0	0
19	0	0	0	0	0	0
20	0	0	1	0	0	0
21	0	0	0	0	0	0
22	0	0	0	0	0	0
23	0	0	0	0	0	1
24	0	0	0	0	0	0
25	0	0	0	0	0	0
26	0	0	0	0	0	0
27	0	0	0	0	1	0
28	0	0	0	0	0	0
29	0	0	1	0	1	1
30	0	0	0	0	0	0
31	1	2	1	0	1	1
32	2	0	0	0	3	1
33	0	1	0	0	1	1
34	1	1	1	1	5	4
35	3	2	3	0	4	4
36	3	5	4	2	5	6
37	5	5	6	2	7	7
38	7	6	5	4	3	7
39	6	3	5	1	5	6
40	5	1	5	2	4	6

Captions for supplementary video

Video S1.
It consists of 7 parts:

Part 1. Grasshopper's escape in response to the robot's forelimb expansion.
High-speed video (960 fps) showing a grasshopper's escape behavior in response to the flush-display by the robot. The escape happens during the forelimbs' spreading stage of the flush-display movement. The white arrow indicates the grasshopper's location before the jump.

Part 2. Grasshopper's escape in response to the robot's forelimb folding movement.
High-speed video (960 fps) showing a grasshopper's escape behavior in response to the flush-display by the robot. The escape happens during the forelimb's folding stage of the flush-display movement. The white indicates the grasshopper's location before the jump.

Part 3. Grasshopper's escape in response to the robot's tail movement.
High-speed video (960 fps) showing a grasshopper's escape behavior in response to the robot's tail-upward movement. The white arrow indicates the grasshopper's location before the jump.

Part 4. Computer animation of the forelimb movement with proto-wings.

Part 5. Computer animation of the forelimb movement without proto-wings.

Part 6. Slow-motion movie of the robot's forelimb movement.
High-speed video (960 fps) of the robot's forelimb movements, starting from the resting position and ending with the positions of the maximal values of the joint angles (as defined in Fig. 3G). The flickering in the video is caused by artificial light pulsating at 60 Hz.

Part 7. Looming circle stimulus used in the neurophysiological experiments.

References cited in supplementary material

1. Collar, N., Christie, D. A., Kirwan, G. M. & del Hoyo, J. Scaly Thrush (*Zoothera dauma*), version 1.0. In Birds of the World (S. M. Billerman, B. K. Keeney, P. G. Rodewald, and T. S. Schulenberg, Editors). Cornell Lab Ornithol. Ithaca, NY, USA. *Cornell Lab of Ornithology, Ithaca, NY, USA* (2020) doi:https://doi.org/10.2173/bow.scathr8.01.
2. Tinbergen, N. *The herring gull's world*. (New York: Basic Books, Inc., 1960).
3. Catania, K. C. Worm grunting, fiddling, and charming - Humans unknowingly mimic a predator to harvest bait. *PLoS One* **3**, (2008).
4. Park, J., Lee, S. & Jablonski, P. G. Unpublished data.
5. Maclean, G. L. & Kirwan, G. M. Egyptian Plover (*Pluvianus aegyptius*), version 1.0. In Birds of the World (J. del Hoyo, A. Elliott, J. Sargatal, D. A. Christie, and E. de Juana, Editors). *Cornell Lab of Ornithology, Ithaca, NY, USA* (2020) doi:10.2173/bow.egyplo1.01.
6. Debus, S. & Kirwan, G. M. Black-breasted Buttonquail (*Turnix melanogaster*), version 1.0. In Birds of the World (J. del Hoyo, A. Elliott, J. Sargatal, D. A. Christie, and E. de Juana, Editors). *Cornell Lab of Ornithology, Ithaca, NY, USA* (2020) doi:10.2173/bow.blbbut2.01.
7. Hughes, J. M. Greater Roadrunner (*Geococcyx californianus*), version 1.0. In Birds of the World (A. F. Poole, Editor). *Cornell Lab of Ornithology, Ithaca, NY, USA* (2020) doi:10.2173/bow.greroa.01.
8. Maxon, M. A. *The real roadrunner*. (University of Oklahoma Press, 2005).
9. Varghese, A. P. Ecology and behaviour of Malabar Trogon Harpactes fasciatus Malabaricus. (Mahatma Gandhi University, 2002).
10. Gill, B. No TitleRifleman (*Acanthisitta chloris*), version 1.0. In Birds of the World (J. del Hoyo, A. Elliott, J. Sargatal, D. A. Christie, and E. de Juana, Editors). *Cornell Lab of Ornithology, Ithaca, NY, USA* (2020) doi:10.2173/bow.riflem1.01.
11. Farnsworth, A. & Lebbin, D. J. Tawny-breasted Flycatcher (*Myiobius villosus*), version 1.0. In Birds of the World (J. del Hoyo, A. Elliott, J. Sargatal, D. A. Christie, and E. de Juana, Editors). *Cornell Lab of Ornithology, Ithaca, NY, USA* (2020) doi:10.2173/bow.tabfly1.01.
12. Munoz Z., J. & Colorado Z., G. J. Importance of tropical mixed-species flocks for migratory birds in shade-grown coffee: implications of foraging together. *J. F. Ornithol.* **92**, 212–230 (2021).
13. Fitzpatrick, J. W., Christie, D. A., Juana, E. de & Kirwan, G. M. Golden-faced Tyrannulet (*Zimmerius chrysops*), version 1.0. In Birds of the World (J. del Hoyo, A. Elliott, J. Sargatal, D. A. Christie, and E. de Juana, Editors). *Cornell Lab of Ornithology, Ithaca, NY, USA* (2020) doi:10.2173/bow.goftyr1.01.
14. Martínez, A. E. & Robinson, S. K. Using foraging ecology to elucidate the role of species interactions in two contrasting mixed-species flock systems in northeastern Peru. *Wilson J. Ornithol.* **128**, 378–390 (2016).
15. Willis, E. O. . The behavior of plain-brown woodcreepers, *Dendrocincla fuliginosa*. *Wilson Ornithol. Soc.* **84**, 377–420 (1972).
16. Boles, W. White-browed Fantail (*Rhipidura aureola*), version 1.0. In Birds of the World (J. del Hoyo, A. Elliott, J. Sargatal, D. A. Christie, and E. de Juana, Editors). *Cornell Lab of Ornithology, Ithaca, NY, USA* (2020) doi:10.2173/bow.whbfan2.01.

17. Boles, W. Willie-wagtail (*Rhipidura leucophrys*), version 1.0. In Birds of the World (J. del Hoyo, A. Elliott, J. Sargatal, D. A. Christie, and E. de Juana, Editors). *Cornell Lab of Ornithology, Ithaca, NY, USA* (2020) doi:10.2173/bow.wilwag1.01.
18. Moeliker, K. Blue-headed Crested-Flycatcher (*Trochocercus nitens*), version 1.0. In Birds of the World (J. del Hoyo, A. Elliott, J. Sargatal, D. A. Christie, and E. de Juana, Editors). *Cornell Lab of Ornithology, Ithaca, NY, USA* (2020) doi:10.2173/bow.bhcfly1.01.
19. Coates, B. J., Dutson, G. C. L. & Filardi, C. E. Family Monarchidae (Monarch-Flycatchers). in *Handbook of the birds of the world. Vol. 11. Old World Flycatchers to Old World Warblers. Barcelona: Lynx Editions.* (eds. del Hoyo, J., Elliott, A. & Christie, D. A. eds.) 244–329 (2006).
20. Boles, W. Yellow-bellied Fairy-Fantail (*Chelidorhynx hypoxanthus*), version 1.0. In Birds of the World (J. del Hoyo, A. Elliott, J. Sargatal, D. A. Christie, and E. de Juana, Editors). *Cornell Lab of Ornithology, Ithaca, NY, USA* (2020) doi:10.2173/bow.yebfan1.01.
21. Clement, P. African Blue Flycatcher (*Elminia longicauda*), version 1.0. In Birds of the World (J. del Hoyo, A. Elliott, J. Sargatal, D. A. Christie, and E. de Juana, Editors). *Cornell Lab of Ornithology, Ithaca, NY, USA* (2020) doi:10.2173/bow.afbfly1.01.
22. Gosler, A. & Clement, P. Yellow-browed Tit (*Sylviparus modestus*), version 1.0. In Birds of the World (J. del Hoyo, A. Elliott, J. Sargatal, D. A. Christie, and E. de Juana, Editors). *Cornell Lab of Ornithology, Ithaca, NY, USA* (2020) doi:10.2173/bow.yebtit3.01.
23. Clement, P. Chestnut-capped Flycatcher (*Erythrocercus mccallii*), version 1.0. In Birds of the World (J. del Hoyo, A. Elliott, J. Sargatal, D. A. Christie, and E. de Juana, Editors). *Cornell Lab of Ornithology, Ithaca, NY, USA* (2020) doi:10.2173/bow.chcfly1.01.
24. Jablonski, P. G. Dark habitats and bright birds: warblers may use wing patches to flush prey. *Oikos* **75**, 350–352 (1996).
25. Marchetti, K. & Trevor, P. The adaptive significance of colour patterns in the old world leaf warblers, genus *Phylloscopus*. *OIKOS* **79**, 410–412 (1997).
26. Clement, P., J., Hoyo, D., Collar, N., Kirwan, G. M. & Christie, D. A. Common Chiffchaff (*Phylloscopus collybita*), version 1.0. In Birds of the World (S. M. Billerman, B. K. Keeney, P. G. Rodewald, and T. S. Schulenberg, Editors). *Cornell Lab of Ornithology, Ithaca, NY, USA* (2020) doi:10.2173/bow.comchi1.01.
27. Fishpool, L. & Tobias, J. A. Fischer's Greenbul (*Phyllastrephus fischeri*), version 1.0. In Birds of the World (J. del Hoyo, A. Elliott, J. Sargatal, D. A. Christie, and E. de Juana, Editors). *Cornell Lab of Ornithology, Ithaca, NY, USA* (2020) doi:10.2173/bow.fisgre1.01.
28. Fishpool, L. & Tobias, J. A. Northern Brownbul (*Phyllastrephus strepitans*), version 1.0. In Birds of the World (J. del Hoyo, A. Elliott, J. Sargatal, D. A. Christie, and E. de Juana, Editors). *Cornell Lab of Ornithology, Ithaca, NY, USA* (2020) doi:10.2173/bow.norbro1.01.
29. Svensson, L. Upcher's Warbler (*Hippolais languida*), version 1.0. In Birds of the World (J. del Hoyo, A. Elliott, J. Sargatal, D. A. Christie, and E. de Juana, Editors). *Cornell Lab of Ornithology, Ithaca, NY, USA* (2020) doi:10.2173/bow.upcwar1.01.
30. Ryan, P. Chestnut-throated Apalis (*Apalis porphyrolaema*), version 1.0. In Birds of the World (J. del Hoyo, A. Elliott, J. Sargatal, D. A. Christie, and E. de Juana, Editors). *Cornell Lab of Ornithology, Ithaca, NY, USA* (2020) doi:10.2173/bow.chtapa3.01.
31. Ryan, P. Black-collared Apalis (*Oreolais pulcher*), version 1.0. In Birds of the World (J. del Hoyo, A. Elliott, J. Sargatal, D. A. Christie, and E. de Juana, Editors). *Cornell Lab of Ornithology, Ithaca, NY, USA* (2020) doi:10.2173/bow.blcapa2.01.

32. Ryan, P. Cricket Longtail (*Spiloptila clamans*), version 1.0. In Birds of the World (J. del Hoyo, A. Elliott, J. Sargatal, D. A. Christie, and E. de Juana, Editors). *Cornell Lab of Ornithology, Ithaca, NY, USA* (2020) doi:doi.org/10.2173/bow.crilon1.01.
33. Madge, S. Common Tailorbird (*Orthotomus sutorius*), version 1.0. In Birds of the World (J. del Hoyo, A. Elliott, J. Sargatal, D. A. Christie, and E. de Juana, Editors). *Cornell Lab of Ornithology, Ithaca, NY, USA* (2020) doi:10.2173/bow.comtai1.01.
34. Kirwan, G. M., Collar, N., Robson, C. & Boesman, P. F. D. Scaly-breasted Cupwing (*Pnoepyga albiventer*), version 2.0. In Birds of the World (N. D. Sly, Editor). *Cornell Lab of Ornithology, Ithaca, NY, USA* (2022) doi:10.2173/bow.sbwbab1.02.
35. Harrap, S. Velvet-fronted Nuthatch (*Sitta frontalis*), version 1.0. In Birds of the World (J. del Hoyo, A. Elliott, J. Sargatal, D. A. Christie, and E. de Juana, Editors). *Cornell Lab of Ornithology, Ithaca, NY, USA* (2020) doi:10.2173/bow.vefnut1.01.
36. Kershner, E. L. & Ellison, W. G. Blue-gray Gnatcatcher (*Polioptila caerulea*), version 1.0. In Birds of the World (A. F. Poole, Editor). *Cornell Lab of Ornithology, Ithaca, NY, USA* (2020) doi:10.2173/bow.buggna.01.
37. Hailman, J. P. A field study of the mockingbird's wing-flashing behavior and its association with foraging. *Wilson Ornithol. Soc.* **72**, 346–357 (1960).
38. Randler, C. Is tail wagging in white wagtails, *Motacilla alba*, an honest signal of vigilance? *Anim. Behav.* **71**, 1089–1093 (2006).
39. Mumme, R. L. Scare tactics in a neotropical warbler: White tail feathers enhance flush-pursuit foraging performance in the slate-throated redstart (*Myioborus miniatus*). *Auk* **119**, 1024–1035 (2002).
40. Jablonski, P. G. & Lee, S. Painted redstarts (*Myioborus Pictus*) attack larger prey when using flush-pursue strategy. *Open Ornithol. J.* **11**, 34–38 (2018).
41. Jablonski, P. G. & McInerney, C. Prey escape direction is influenced by the pivoting displays of flush-pursuing birds. *Ethology* **111**, 381–396 (2005).
42. Jablonski, P. G. A rare predator exploits prey escape behavior: The role of tail-fanning and plumage contrast in foraging of the painted redstart (*Myioborus pictus*). *Behav. Ecol.* **10**, 7–14 (1999).
43. Hilbie, C. & Block, N. L. Collared Redstart (*Myioborus torquatus*), version 1.0. In Birds of the World (T. S. Schulenberg, Editor). *Cornell Lab Ornithol. Ithaca, NY, USA* (2020) doi:10.2173/bow.colred1.01.
44. Senter, P. & Robins, J. H. Range of motion in the forelimb of the theropod dinosaur Acrocanthosaurus atokensis, and implications for predatory behaviour. *J. Zool.* **266**, 307–318 (2005).
45. Senter, P. Comparison of forelimb function between *Deinonychus* and *Bambiraptor* (Theropoda: Dromaeosauridae). *J. Vertebr. Paleontol.* **26**, 897–906 (2006).
46. Senter, P. & Robins, J. H. Resting orientations of dinosaur scapulae and forelimbs: A numerical analysis, with implications for reconstructions and museum mounts. *PLoS One* **10**, 1–21 (2015).
